# Fast-Dissolving and Highly Soluble Dasatinib Multicomponent Crystals Efficiently Inhibit Leukemia Cell Viability

**DOI:** 10.1021/acsomega.6c01923

**Published:** 2026-09-19

**Authors:** Luan F. Diniz, Luiz C. G. Diógenes, Paulo S. Carvalho Jr, Ezequiel A. D. Junior, Alexia D. Nascimento, Maria E. Cortés, Rubén Dario Sinisterra, Renata Diniz, Christian Fernandes

**Affiliations:** † Departamento de Produtos Farmacêuticos, Faculdade de Farmácia, 28114 Universidade Federal de Minas Gerais, 31270-901 Belo Horizonte, MG, Brazil; ‡ Instituto de Física, 54534 Universidade Federal do Mato Grosso do Sul, 79074-460 Campo Grande, MS, Brazil; § Departamento de Odontologia Restauradora, Faculdade de Odontologia, Universidade Federal de Minas Gerais, 31270-901 Belo Horizonte, MG, Brazil; ∥ Inovação Tecnológica, Instituto de Ciências Exatas, Universidade Federal de Minas Gerais, 31270-901 Belo Horizonte, MG, Brazil; ⊥ Departamento de Química, Instituto de Ciências Exatas (ICEx), Universidade Federal de Minas Gerais, 31270-901 Belo Horizonte, MG, Brazil

## Abstract

Dasatinib (DAS) is a biopharmaceutical classification system (BCS) class II drug that is used orally to treat chronic myelogenous leukemia. However, its very low and pH-dependent aqueous solubility severely limits its oral bioavailability. To address this biopharmaceutical limitation, we designed more soluble multicomponent crystalline forms (MCCs) of DAS. Two novel salt solvate forms were successfully obtained via solvent evaporation through reactions with maleic and fumaric acids. A comprehensive solid-state evaluation of these salt solvate forms was conducted by using X-ray diffraction (SCXRD, PXRD), spectroscopy (FT-IR and ^1^H NMR), and thermal (TG and DSC) analyses. Both MCCs of DAS have displayed substantial enhancements in buffered aqueous solubility (up 1470-fold) and intrinsic dissolution rate (up 312-fold) compared with the parent drug. Furthermore, the DAS MCCs exhibited an increased cytotoxicity and selectivity toward tumor cells. These fulfillments highlight the potential of crystalline salt engineering to enhance the unsatisfactory oral bioavailability and anticancer efficacy of the DAS.

## Graphical abstract

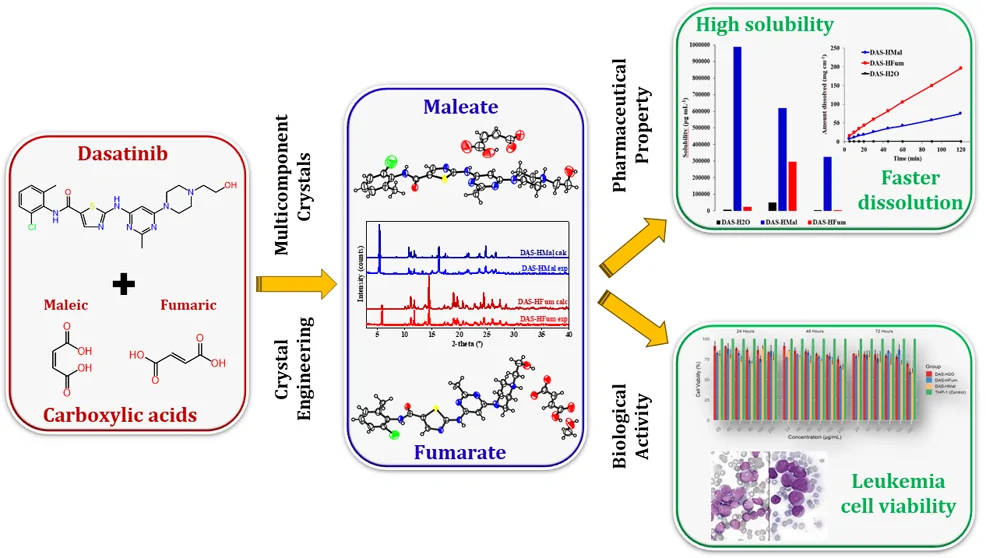

## 1 Introduction

Crystal-engineered multicomponent pharmaceutical crystals have become a consolidated, highly effective, and cost-effective approach for enhancing the active pharmaceutical ingredients (APIs).^1–4^ Its extensive application in developing innovative APIs and repositioning of older ones has allowed this approach to gain widespread and unanimous acceptance in the pharmaceutical field.^5–8^ Creating custom crystalline salt structures by incorporating API-complementary counterion into the crystal lattice while maintaining covalent bonds ensures the drug’s pharmacological activity and provides a pathway for optimizing its physicochemical properties, mainly aqueous solubility and dissolution rate.^9–13^ For basic, ionizable, and weakly soluble APIs, such as dasatinib (DAS, Scheme 1), pharmaceutically acceptable carboxylic acids are typically utilized for drug protonation, turning it into novel soluble salts.^14^

**Scheme 1:**
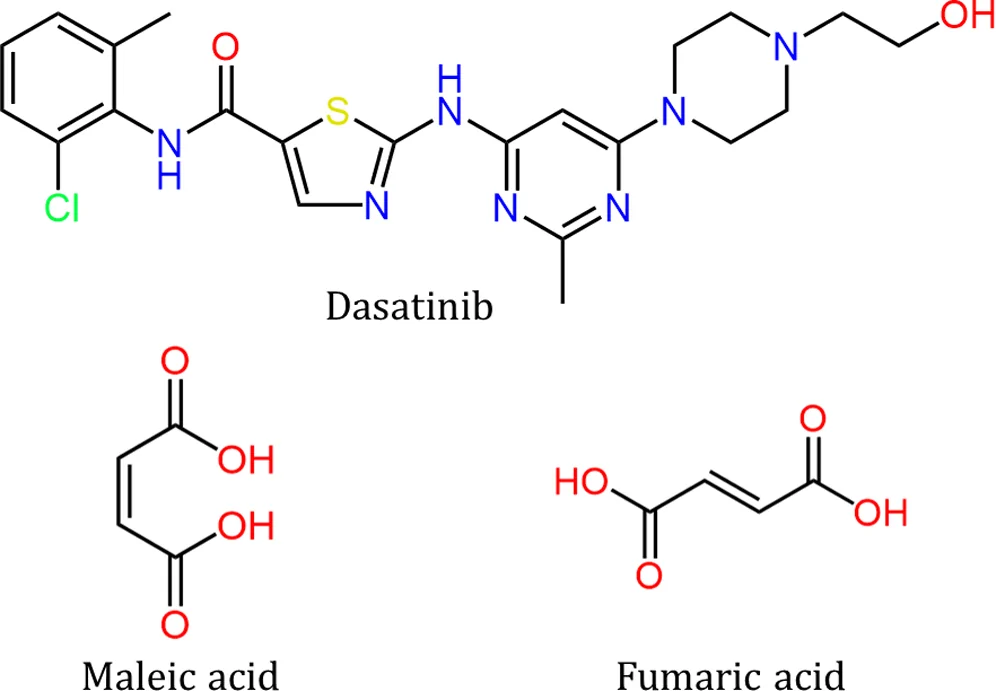
Molecular Structure of Dasatinib (DAS) and the Coformers (Maleic and Fumaric Acids)

DAS is a Biopharmaceutical Classification System (BCS) class II drug, which is orally active. It is a multitargeted tyrosine kinase inhibitor used in the treatment of chronic myelogenous leukemia.^15^ Although DAS is ionizable, this API is marketed in its neutral form, exhibiting low and pH-dependent aqueous solubility as well as a slow dissolution rate.^16^ Consequently, DAS has a poor oral bioavailability of 14%.^17^ Also, its very short half-life of 3 to 5 h requires frequent dosing, which affects patient compliance due to the occurrence of systemic side effects.^18^ To overcome these biopharmaceutical issues, several approaches have been employed to date, including salt solvate crystallization,^19,20^ cyclodextrin complexation,^21^ nanocrystal-based formulations,^22^ amorphous solid dispersions,^23^ self-nanoemulsifying systems,^24^ polymeric micelles,^25^ and hybrid exosomes.^26^ For DAS, these investigations have demonstrated that significantly enhancing the drug’s solubility and dissolution rate positively benefits its pharmacokinetic profile.

From a crystal engineering perspective, prior studies on DAS have concentrated on describing the solid-state structure of multicomponent crystals (MCCs) without an accompanying proper pharmaceutical evaluation. Sarceviča et al.^27^ and Trimdale-Deksne et al.^28^ successfully determined the crystal structures of twenty-three DAS solvates formed with various organic solvents. These solvents included alcohols such as isopropanol, n-butanol, *n*-pentanol, and isoamyl alcohol, along with dimethyl sulfoxide, dimethylformamide, ketones like cyclohexanone and 4-methyl-2-pentanone, esters, chloroform, and nitromethane, among others. They classified the solvates into three structural packing groups based on molecular conformation, hydrogen bonding patterns, and layer arrangements but reported no solubility or dissolution data. Additionally, three recent publications^19,20,29^ described DAS MCCs with carboxylic acid coformers, although only one of these is an unambiguously confirmed salt. Mondal et al.^20^ reported dasatinib succinate hydrate, confirming the protonation of DAS through single-crystal X-ray diffraction. They also investigated dasatinib-l-glutamic acid and dasatinib-4-hydroxybenzoic acid MCCs, although the ionization states of these were not established crystallographically. The other two studies, by Abdul Azeeze et al.^19^ and Saladi et al.,^29^ respectively, reported assumed cocrystals (with 4-hydroxybenzoic acid, 4-chlorobenzoic acid, and sorbic acid) and solvates (with hexanoic, heptanoic, and octanoic fatty acids).

While these cited studies provided adequate solid-state characterization, only one of them has undoubtedly elucidated the crystal structure of the only known DAS salt to date (succinate hydrate). Regarding biopharmaceutical performance, the available solubility and dissolution data for DAS in scientific reports are incomplete and do not adequately address conditions relevant to physiological processes. For these reasons, our work is unique. It stands out from recent studies of DAS cocrystallization because it elucidates salt solvate crystal structures and characterizes them energetically and conformationally, unlike prior reports. In addition, it carefully outlines the API biopharmaceutical profile under conditions that closely resemble physiological conditions. Finally, it evaluates the cytotoxicity and selectivity toward tumor cells.

Given the poor water solubility and slow dissolution rate of DAS, which considerably restrict its oral bioavailability, we have focused on an effective crystal engineering strategy to develop two highly soluble and faster-dissolving salt solvates, aiming to enhance the biopharmaceutical profile of DAS. These salts solvated have been obtained from the drug’s reaction with pharmaceutically acceptable acids, maleic and fumaric. Besides a thorough solid-state characterization by single-crystal and powder X-ray diffraction (SCXRD and PXRD), energy framework (EF) calculations, Fourier-transform infrared (FT-IR) spectroscopy, proton nuclear magnetic resonance (^1^H NMR), thermogravimetry (TG), and differential scanning calorimetry (DSC), we also performed equilibrium solubility as well as dissolution studies. A cytotoxicity evaluation employing the human leukemia cell line THP-1 was conducted to reveal the enhanced anticancer activity of the new DAS MCCs. All outcomes acquired provide vital insights into the oncological use of DAS, as its solvate salts have been proven to be biopharmaceutically advantageous compared to the parent DAS form.

## 2 Materials and Methods

### 2.1 Materials

Dasatinib (purity >99%), organic solvents (analytical, spectroscopic, or chromatographic grade), and the coformers (maleic and fumaric acids) were purchased from Sigma-Aldrich Brazil and used as received. Ultrapure water was obtained from a Millipore Direct-Q3 system and was used directly.

### 2.2 Preparation of DAS Solid Forms

*Dasatinib fumarate salt solvate* (*DAS-HFum-sol*): 200 mg of DAS (0.498 mmol) and 50 mg of fumaric acid (0.498 mmol) were manually ground in a pestle mortar until a homogeneous mass was formed by adding 2 mL of hot methanol. The resulting ground material was redissolved in 10 mL of hot methanol for recrystallization. Suitable colorless plate crystals of DAS-HFum-sol were observed within 1–2 days by slow evaporation of solvent at room temperature.

*Dasatinib maleate salt solvate* (*DAS-HMal-sol*): 200 mg of DAS (0.498 mmol) and 50 mg of maleic acid (0.498 mmol) were mechanochemically reacted in a pestle mortar by adding 2 mL of hot ethanol until a homogeneous system was formed. Suitable plate crystals of DAS-HMal-sol appeared within 1–2 days by recrystallizing this material in 10 mL of hot ethanol, maintained under slow evaporation at room temperature.

### 2.3 X-ray Diffraction Analysis

Single-crystal X-ray diffraction (SCXRD) data were collected at room temperature (298 ± 2 K) on a Rigaku XtaLAB Synergy diffractometer with a HyPix detector system using a Cu (λ = 1.54187 Å) microfocus radiation source. The acquisition, indexing, integration of the reflections, and unit cell determinations were performed with CrysAlisPro software. Using Olex2,^30^ the structures were solved by intrinsic phasing with SHELXT^31^ and refined by full-matrix least-squares minimization with SHELXL.^32^ The non-hydrogen atoms were refined anisotropically. All hydrogen atoms were located from electron-density difference maps and were positioned geometrically and refined using the riding model [U_iso_(H) = 1.2*U*_eq_ or 1.5*U*_eq_]. PLATON/SQUEEZE was used to remove disordered solvent.^33^ Crystallographic representations have been created through the MERCURY 4.3 ^34^ and CrystalExplorer 21.5 ^35^ programs. All *CIF* files are available at www.ccdc.cam.ac.uk under the CCDC^36^ numbers 2528707 and 2528708.

Powder X-ray diffraction (PXRD) data were acquired at room temperature on an XRDynamic 500 Anton Paar diffractometer, operating at 40 kV of voltage and 50 mA of current, in a Bragg–Brentano geometry and using CuKα radiation (λ = 1.54187 Å). The diffractograms were collected over an angular range of 3–45° (2θ) with a step size of 0.02° (2θ) and a constant counting time of 20 s per step.

### 2.4 Energy Framework Calculations

The energy frameworks (EFs) were computed based on density functional theory (B3LYP/6–31G­(d,p)) molecular wave function using CrystalExplorer 21.5^35^ software and *CIF* files as input. The total intermolecular interaction energy (*E*_tot_) is given as the sum of the electrostatic (*E*_ele_), polarization (*E*_pol_), dispersion (*E*_dis_), and exchange–repulsion (*E*_rep_) components, with scale factors of 1.057, 0.740, 0.871, and 0.618, respectively. Intermolecular interactions between two adjacent molecules were ignored when the closest interatomic distance was higher than 3.8 Å. The resulting EFs were graphed by linking the centers of mass of molecules so that the cylinder thicknesses are set in proportion to the magnitude of the intermolecular interaction energies.

### 2.5 Spectroscopy Analysis

Fourier transform infrared (FT-IR) spectra were recorded on a PerkinElmer Spectrum One spectrometer equipped with an attenuated total reflectance (ATR) device. In measurements, 64 scans over a range of 4000–650 cm^–1^ were collected with a spectral resolution of 4 cm^–1^.

The ^1^H NMR data were obtained at room temperature on a Bruker Avance DRX400 spectrometer, operating at 400 MHz, with a spectrum resolution of 0.12 Hz, a zg 30 pulse program with ns 16, d1 1s, an acquisition time of 4.09 s, and a spectral width of 20 ppm. For the acquisition and processing of ^1^H NMR spectra, the TopSpin 4.0 software package was used. The phase and baseline were manually corrected, and the TMS signals were calibrated at 0.00 ppm. Proton chemical shifts (δ) were given in ppm and coupling constants (*J*) in Hz. For the NMR data collection, approximately 10 mg of DAS samples was dissolved in 600 μL of DMSO-*d*_6_ and transferred to NMR tubes.

### 2.6 Thermal Analysis

Thermogravimetric (TG) analysis was carried out using a Shimadzu TGA-60 thermobalance. Approximately 5.0 mg of the samples was placed in open alumina pans and heated under N_2_ flow (50 mL min^–1^) from 25 to 500 °C at a heating rate of 10 °C min^–1^. Differential scanning calorimetry (DSC) curves were acquired using a Shimadzu DSC-60 instrument. The samples (2.0 ± 0.2 mg) were placed in open aluminum pans and heated under a N_2_ atmosphere (50 mL min^–1^ at a heating rate of 10 °C) until the degradation temperature of each DAS sample. The obtained thermal data were analyzed and plotted using the Shimadzu TA-60 software.

### 2.7 Ultra-Performance Liquid Chromatography

The quantitative analysis of DAS solid forms from solubility, dissolution, and stability studies was conducted on a Waters Acquity UPLC system equipped with a diode array detector (DAD). Empower 3.0 software was used for the acquisition and analysis of chromatograms. A Zorbax Eclipse Plus C18 column (100 mm length × 2.1 mm i.d., 1.8 μm particle size), maintained at 35 °C, was used for separation. The mobile phase consisted of a mixture of ammonium acetate buffer pH 4.0 and acetonitrile (75:25, v/v), delivered at a flow rate of 0.6 mL min^–1^. DAS was detected at 323 nm, and the injection volume was 5 μL. The method validation followed the ICH Q2 (R2) guideline.^37^ The parameters selectivity, linearity, precision, accuracy, and limits of quantitation and detection were evaluated, and the main results are summarized in Table S1.

### 2.8 Equilibrium Solubility and Intrinsic Dissolution

Equilibrium solubilities of DAS solid forms were determined through the shake-flask method^38^ at 37 ± 0.5 °C in five dissolution media: 0.01 M HCl solution (pH 2.0), acetate buffer (pH 3.6), acetate buffer (pH 4.5), phosphate buffer (pH 5.4), and phosphate buffer (pH 6.8). Suspensions were prepared in triplicate by stirring at 150 rpm an excess of DAS solid phases into 2 mL of each dissolution medium, being maintained at 150 rpm for 24 h to ensure saturation. Afterward, the suspensions were filtered through a 0.45 μm syringe filter and diluted in their respective dissolution media before being quantified by UPLC-DAD. Upon completing the equilibrium solubility tests, the solid sediment’s identity was confirmed through PXRD analysis, and the pH of each dissolution medium was measured using a pH meter.

Intrinsic dissolution experiments were conducted in triplicate using a rotating disk dissolution apparatus. Approximately 200 mg of each DAS solid form was compressed with a hydraulic pump at 1 kN for 1 min to create nonporous and compact disks measuring 0.5 cm^2^, with one side featuring a flat surface. The disk attachments were rotated at 100 rpm while submerged in 500 mL of acetate buffer at pH 3.6, preheated to 37.0 ± 0.5 °C. At specific time intervals (5, 10, 15, 20, 30, 45, 60, 90, and 120 min), 5 mL of dissolution medium was withdrawn and filtered through a 0.45 μm syringe filter. The concentration of dissolved DAS was then measured using UPLC-DAD. Any possible phase transitions during the dissolution experiments and disk compression were monitored through PXRD from the undissolved solids.

### 2.9 Stability Studies

DAS solid forms were exposed under accelerated degradation conditions specified by ICH, i.e., at 40 °C and 75% relative humidity (RH), for 180 days to evaluate the drug’s solid-state stability. To achieve these conditions, a calibrated climate chamber, model 420/CLD-300 from Nova Ética, has been employed. At the same time, the photostability of DAS samples was checked through a photodegradation test. Irradiation of samples was conducted at 20 °C for 5 days in a photostability chamber EF/0,2/R-F-220 Mecalor equipped with UV and visible lamps, achieving a light intensity of 1.2 million lux h^–1^ (visible) and 200 W h m^–2^ (UV), as recommended by ICH guidance.^39^ In both stability assessments, the DAS content has been confirmed by UPLC-DAD evaluations.

### 2.10 Cell Lines and Culture Conditions

Fibroblasts L929 and human leukemia THP-1 cell lines were grown in cell culture flasks containing Dulbecco’s modified Eagle’s medium (DMEM) and Roswell Park Memorial Institute medium (RPMI), respectively, both containing high glucose (4500 mg L^–1^), supplemented with 10% fetal bovine serum (FBS), 1% antibiotic/antimycotic (penicillin 10,000 units mL^–1^/streptomycin 10 mg mL^–1^), 1% l-glutamine, and 1% nonessential amino acids. All cells were incubated in a humidified incubator containing 5% CO_2_ and a temperature of 37.5 °C.

### 2.11 *In Vitro* Cytotoxicity Tests

The cell viability of L929 and THP-1 cell lines in indirect contact with DAS solid forms was evaluated by the 3-(4,5-dimethylthiazol-2yl)-2,5-diphenyl tetrazolium bromide (MTT) assay. L929 and THP-1 cells were treated with DAS commercial form (DAS-H_2_O) and DAS-HFum-sol/DAS-HMal-sol salt solvate forms at concentrations of 20, 40, 60, 80, 100, and 200 μg mL^–1^ for 24, 48, and 72 h. Following treatments, cells were incubated with 10 μL of MTT solution at 5 mg/mL, and 90 μL of phenol red-free DMEM was added to each well to form the formazan crystals over 4 h. The absorbance of the formazan crystals, dissolved overnight in sodium dodecyl sulfate (SDS), was measured at 570 nm using a spectrophotometer.

## 3 Results and Discussion

### 3.1 Crystal Structure Description

DAS has several functional groups, enabling the formation of diverse supramolecular motifs. Its molecular structure comprises a pyrimidine–thiazole core attached to a hydroxyl-piperazine and Cl-phenylacetamide moieties, which includes eight hydrogen bond acceptors and three hydrogen bond donors (Scheme 1). The piperazine nitrogen in DAS can participate in proton–acceptor interactions (p*K*_*a*_ = 6.8), as observed by its known salt solvate forms. Salt formation with carboxylic acids is highly expected^40,41^ (Table S2), offering a promising strategy to enhance the DAS solubility and dissolution rate. By enabling ionization and providing hydrogen-bond donors, which otherwise require hydration, this strategy provides a robust alternative to solvation.^41^ DAS has been reported to form numerous solvates and even multicomponent salt forms; nevertheless, the 3D structures of many of these salt solvate systems are rarely known, and the relationship between structure and solubility remains unexplored. Herein, we elucidated the crystal structure of two DAS solvated salts and correlated the relationship between crystallographic features, the impact of coformer molecule insertion, intermolecular interactions, the energy framework, and, most importantly, the drug’s aqueous solubility, dissolution rate, cytotoxicity, and selectivity toward tumor cells.

To further increase the solubility and dissolution rate of DAS, we have prepared MCCs with fumaric and maleic acids, successfully yielding the DAS-HFum-sol and DAS-HMal-sol salt solvates. Toward understanding the structural attributes driving the stabilization of these systems in the solid state, we provide detailed crystallographic evaluations below. Figure S1 illustrates the asymmetric units (ASUs) of DAS salt solvates, whereas Table 1 and S3 summarize the crystallographic data and H-bond geometric parameters, respectively. Figure S2 shows the electron density maps, confirming the N atom protonation site of the DAS molecule and, thereby, the salt formation.

**Table 1:** Crystallographic Data and Refinement Parameters of DAS MCCs

identification code	DAS-HFum-sol	DAS-HMal-sol
empirical formula	C_27_H_34_ClN_7_O_7_S	C_26_H_30_ClN_7_O_6_S
formula weight	636.12	604.08
temperature (K)	298.0 (2)	298.0 (2)
crystal system	monoclinic	monoclinic
space group	*P*2_1_/n	*C*2/c
a (Å)	18.4070(3)	45.209(5)
b (Å)	9.37730(10)	8.4731(4)
c (Å)	18.8052(3)	22.736(2)
α (deg)	90	90
β (deg)	108.149(2)	133.705(18)
γ (deg)	90	90
volume (Å)^3^	3084.44(8)	6296.0(17)
Z/Z′	4/1	8/1
ρ_calc (_g cm^3^)	1.370	1.275
μ (mm^–1^)	2.204	2.110
crystal size/mm^3^	0.12 × 0.09 × 0.02	0.10 × 0.08 × 0.01
reflections collected	42,826	67,118
independent reflections	5633	5549
unique reflections	4915	2864
R_1_ [I ≥ 2σ(I)]	0.0412	0.0638
wR_2_ [all data]	0.1156	0.2120
goodness-of-fit on F^2^	1.046	1.006

#### 3.1.1 Dasatinib Fumarate (DAS-HFum-sol) Salt Solvate

The DAS-HFum-sol crystallizes in the monoclinic space group *P*2_1_/c (Z’ = 1, Table 1) with one ionized DAS^+^ cation, a hydrogen-fumarate anion (HFum^–^), and a methanol solvent molecule in the asymmetric unit (Figure S1a). Methanol acts as a lattice solvent stabilizing the DAS^+^:HFum^–^ pair through hydrogen bonding. We acknowledge that a solvated salt containing methanol might limit its pharmaceutical application. However, the multiple attempts at solvent exchange in the synthetic process of this salt form did not yield a novel DAS MCC, as we had intended. Salt formation occurs through the transfer of a proton from the acid to the piperazine moiety of the DAS molecule. The ionic pair forms through the *R*_2_^2^(8) synthon, which involves the amine-hydroxyl portion of the DAS^+^ cation and the carboxylate moiety of the counterion (Figure 1a). In the salt solvate form, the DAS^+^ cation adopts a conformation in which the aromatic pyrimidine–thiazole core and the mean plane of the pyrimidine ring are 70.9(2)°, oriented relative to each other. The hydroxyl group adopts a synclinal orientation relative to the N atom of the pyrimidine ring (N–C–C–O = −40.2(3)°). Due to steric hindrance, the chlorophenyl ring adopts an almost orthogonal orientation concerning the central aromatic system, leading to syn-periplanar orientation between the amide oxygen and the thiazole sulfur atom (O–C–C–S = 16.7(2)°).

**Figure 1:**
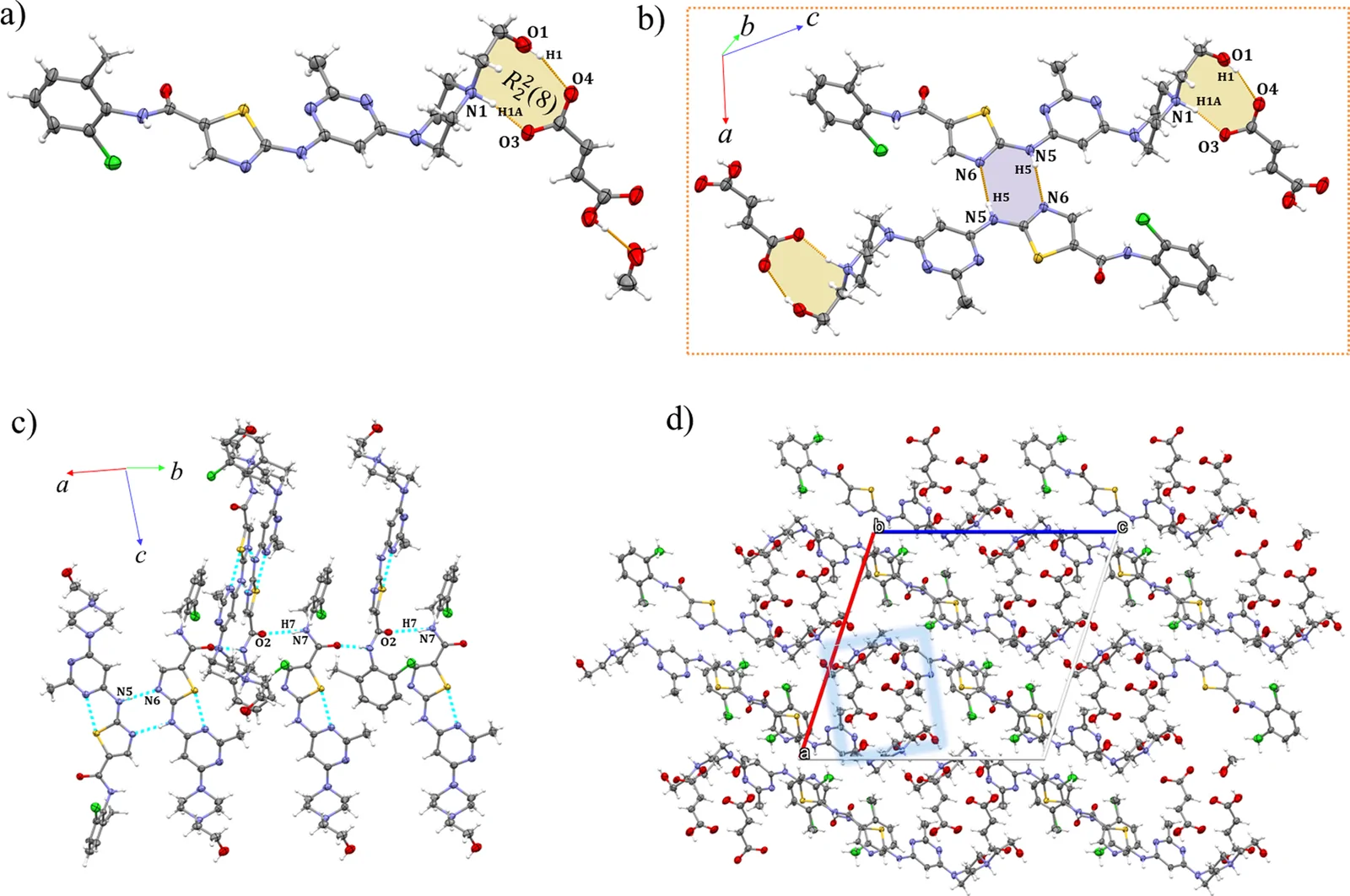
(a) Asymmetric unit view, (b) planar unit from the assembly of ionic pairs, (c) the 2D-sheet motif, and (d) crystal packing of the DAS-HFum-sol salt solvate.

The reported fumarate displays a conformation that differs from its hydrated (commercial form) and succinate salt counterparts.^20,42^ The hydrated and succinate forms exhibit a more extended conformation of the ionized group relative to the central molecular core (see Figure S3). In the DAS-HFum-sol, the ionic pairs are centrosymmetrically linked through a homosynthon involving the thiazole rings of DAS^+^ cations, forming a planar unit (Figure 1b). These dimeric units are further connected via NH•••O H-bonds (N7–H7•••O2, 2.958(2)­Å, 172.09(11)°) between amide groups of adjacent cations, resulting in a 2D sheet extending along the [010] direction. Within this sheet, neighboring planar units are oriented nearly orthogonally, forming an angle of 87.09(2)° between the mean planes of adjacent dimers (Figure 1c). As the anions are located on the exterior of these motifs, adjacent fumarate anions are then bridged by methanol molecules, providing the assembly of the sheets into a 3D structure as shown in Figure 1d. The packing of these 2D sheets leads to the formation of polar domains, enriched by the presence of the anions, whereas the solvent molecules play a crucial role in stabilizing the overall crystal structure.

#### 3.1.2 Dasatinib Maleate (DAS-HMal-sol) Salt Solvate

The structural determination of DAS-HMal-sol presented manifold challenges. Pronounced disorder effects (disordered ethanol solvent molecules and substitutional disorders) needed to be modeled, with only the substitutional disorder being properly treated. In the final refinement model, the disordered ethanol that fills the solvent-accessible channels of the crystal structure has been removed by a SQUEEZE procedure. Despite our repeated efforts to obtain high-quality single crystals, all solids exhibited twinning. An appropriate twin law has been found; nevertheless, the refinement did not converge to an acceptable structural model. Thus, the most reliable and satisfactory model for the DAS-HMal-sol salt solvate is based on the twinned and “squeezed” structure. The identity of the compound was unambiguously confirmed through SCXRD analysis, as shown in its asymmetrical unit representation (Figure S1b). DAS-HMal-sol crystallizes in the monoclinic space group *C*2/*c* with Z’ = 1 (Table 1 and Figure 2a). Interestingly, its unit cell volume is approximately twice that of DAS-HFum-sol. Despite the larger unit cell, the calculated density of DAS-HMal-sol (1.275 g cm^–3^) is slightly lower than that of DAS-HFum-sol (1.370 g cm^–3^), highlighting the difference in crystal packing between the two salt solvates.

**Figure 2:**
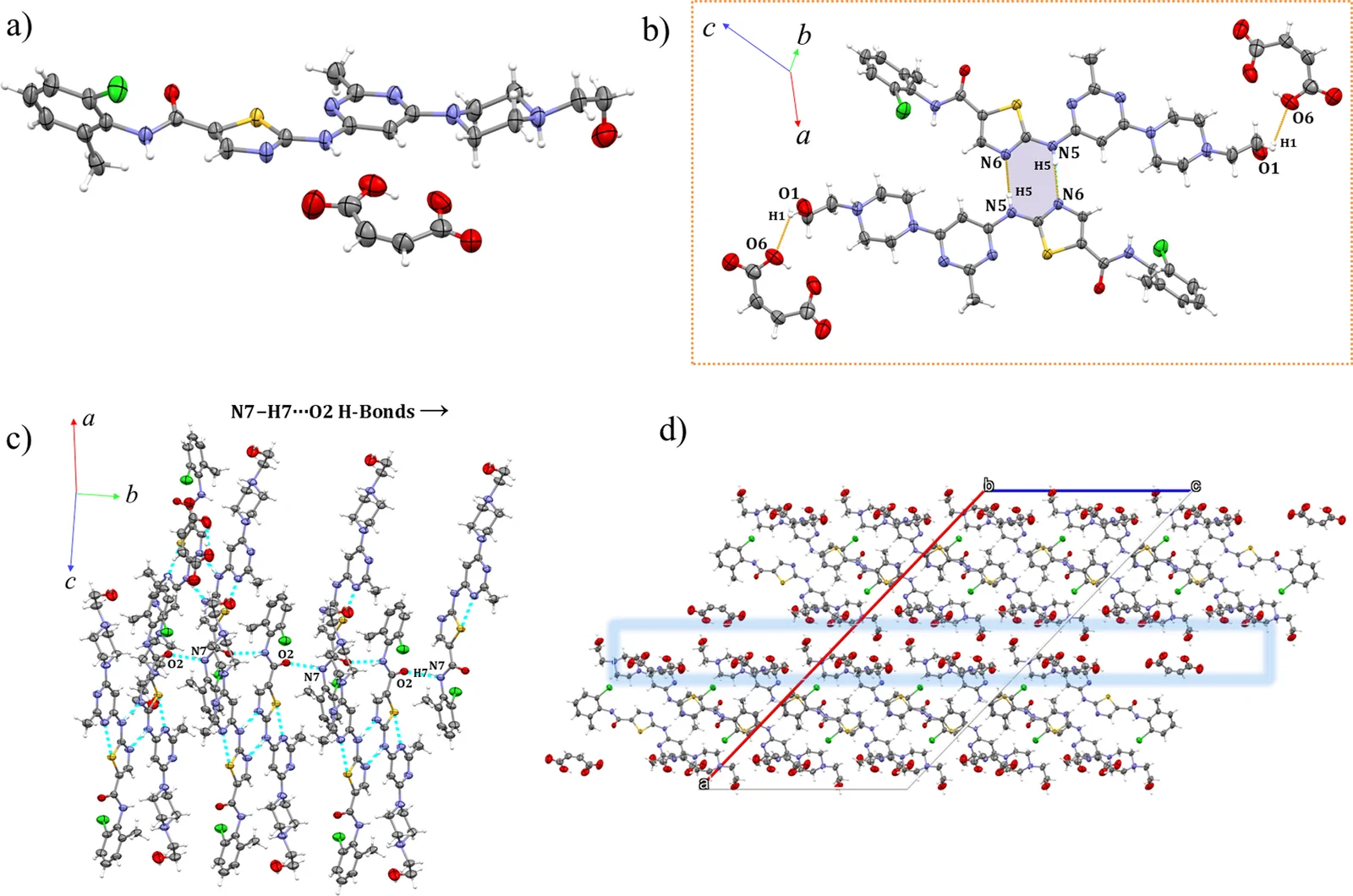
(a) Asymmetric unit view without disordered ethanol (removed by a SQUEEZE procedure), (b) planar unit from the assembly of ionic pairs, (c) the 2D sheet motif, and (d) crystal packing of the DAS-HMal-sol salt solvate.

A notable structural distinction lies in the conformation of the DAS^+^ cation. In the maleate salt solvate, the DAS^+^ cation exhibits an antiorientation of the nitrogen atom from the pyrimidine ring relative to the adjacent carbon bearing the methyl group, with a N–C–C–C torsion angle of 176.7(5)°. Additionally, within the piperazine moiety, the nitrogen and hydroxyl atoms adopt a torsion angle of 51.4°. The conformations of DAS in both the fumarate and maleate salt solvates differ significantly from those observed in the DAS hydrate and succinate salt (Figure S3). Interestingly, in the structure of the DAS-HMal-sol salt solvate, there is no direct hydrogen bonding between the cation and the anion. However, similar to the fumarate salt solvate, ionic pairs assemble into dimeric units through a homosynthon formed by interactions involving the central portion of the cations (Figure 2b). These planar units extend into a 2D sheet via NH•••O H-bonds (N7–H7•••O2, 2.818(5)­Å, 157.67(26)°) along the [010] direction. Within this motif, adjacent dimers are oriented at an angle of 66.01(2)° relative to each other, as measured between their mean planes (Figure 2c). Notably, the anions remain aligned within this motif and do not orient themselves outside of it. The motifs are arranged in an antiparallel manner due to the OH•••COO H-bonds (O1–H1•••O6, 2.853(8)­Å, 137.04(41)°) from adjacent dimers. As a result, the packing of these 2D sheets generates a continuous anionic channel throughout the structure, creating solvent-accessible regions. As shown in Figure 2d, the crystal packing of DAS-HMal-sol gives rise to a significantly more pronounced hydrophilic domain compared to that observed in the fumarate salt solvate.

### 3.2 Infrared Spectroscopy

Infrared spectroscopy has confirmed the proton transfer process and accurately identified the positions of hydrogen atoms within the structures. By offering detailed information on the molecular vibrational modes,^43^ this technique was used in complement to characterize the new DAS salt solvates. Figure 3 presents the FT-IR spectra of both DAS monohydrate (raw material) and its multicomponent solvated salts. Band assignments (Table S4) and spectral interpretation were performed based on the evaluation of salt solvate structures (Section 3.1) and using reference spectroscopic data available for analogous DAS compounds.^19,20,23^ This API exhibits characteristic infrared stretching frequencies at 3205 cm^–1^ (N–H stretch), 1630 cm^–1^ (amide C=O stretch), and 1612 cm^–1^ (C=C stretch).

**Figure 3:**
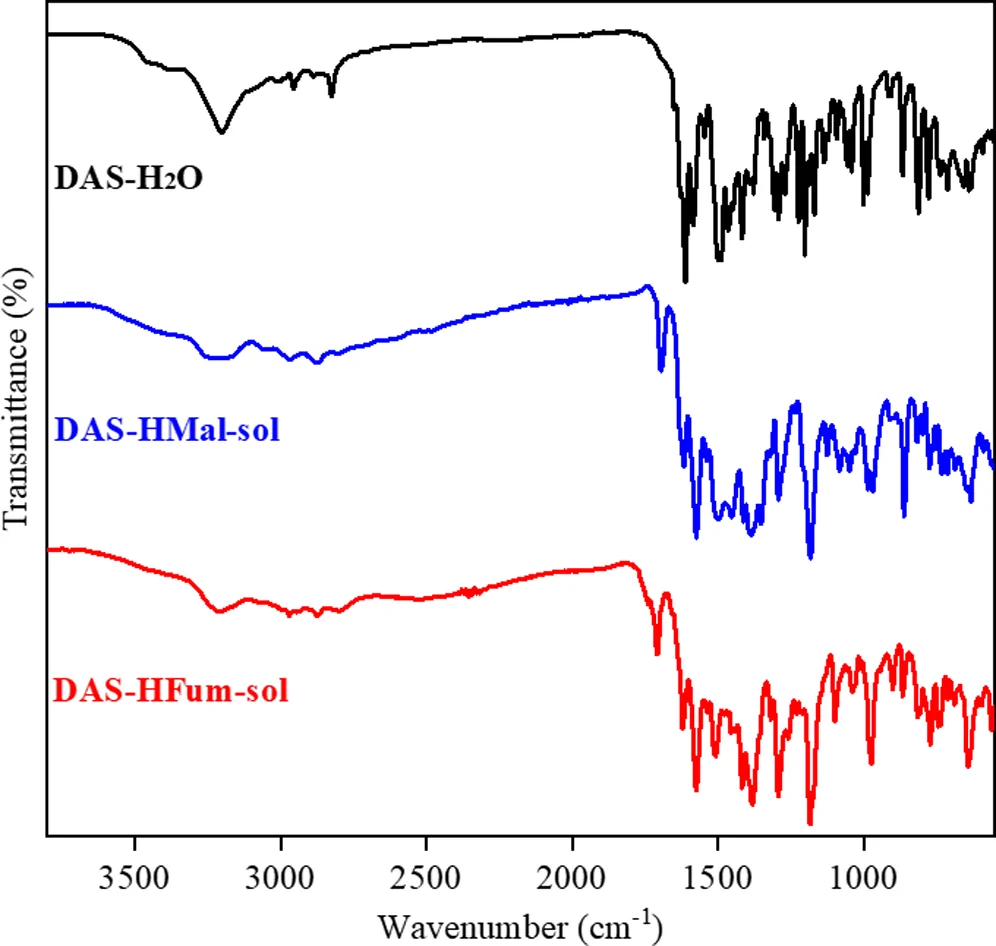
FT-IR spectra of DAS and its new salt solvates.

Salt formation has been confirmed since these typical vibrational modes of the DAS molecule were also observed in the salt FT-IR spectra, and further, the appearance of bands related to the dicarboxylic acids (coformers) was noted. The new absorption bands at approximately 1700 cm^–1^ in the salt FT-IR spectra have been assigned to the acid C=O stretching modes of partially deprotonated hydrogen maleate and hydrogen fumarate anions. Additionally, the salt FT-IR spectra show new bands ranging from 1574 to 1412 cm^–1^, associated with carboxylate antisymmetric and symmetric stretching modes (*COO* ^–^) of the counterion species, confirming the presence of the *COO* ^–^ salt-forming group on the coformer molecules. These observations corroborate the crystallographic analyses regarding the proton transfer and the real H atom positions (Figure S2).

### 3.3 Proton Nuclear Magnetic Resonance

The ^1^H NMR experiments have been used to determine the stoichiometric ratio between DAS and the salt formers by analyzing the integral values of each signal, thereby verifying the sample’s purity in solution. Assignments of the proton chemical shifts and spectra interpretation were carried out using DAS ^1^H NMR spectra reported in the literature.^21,44,45^ The ^1^H NMR spectra of both DAS solvated salts (Figures S4 and S5) exhibited all the signals required for the DAS molecule, besides the expected signals corresponding to the maleate and fumarate anions assigned to the =CH olefinic group at 6.05 and 6.62 ppm. Additionally, the integral values in the ^1^H NMR spectra of the new DAS salts solvated confirm that the crystal structures contain DAS^+^ cation and the corresponding counterion in a 1:1 ratio, attesting to the purity of these crystalline phases in solution. It is worth mentioning that typical signals of methanol and ethanol molecules appeared in the spectra, confirming the solvatomorphic nature of the new DAS MCCs. For ethanol, the characteristic signals were observed at approximately 1.0 ppm (−CH_3_), 3.4 ppm (−CH_2_), and 4.5 ppm (−OH). For methanol, the typical signals were found at around 3.2 ppm (−CH_3_) and 5.0 ppm (−OH), with the last signal being discrete and broad.

### 3.43.4 Powder X-ray Diffraction

Powder X-ray diffraction (PXRD) is an undoubtedly useful characterization tool for demonstrating the formation of multicomponent crystals from a crystallization screening.^46^ Changes in the 2θ peak positions observed in the experimental diffractograms, compared to the starting materials, indicate the establishment of a new and unambiguous crystalline phase. PXRD confirms that the single crystal mounted for the SCXRD data collection is representative of the synthesized bulk sample and is also used herein to check the purity of the DAS salt solvates. First, as presented in Figure 4a, both experimental PXRD patterns of DAS maleate and fumarate are entirely distinct from the DAS raw material. Second, experimental patterns exhibit excellent agreement with the theoretical ones, calculated from the *CIF* files obtained from the refinements. Hence, we confirm that all single crystals formed correspond to pure and unique salt solvate crystalline phases, which also represent the entire synthesized sample.

**Figure 4:**
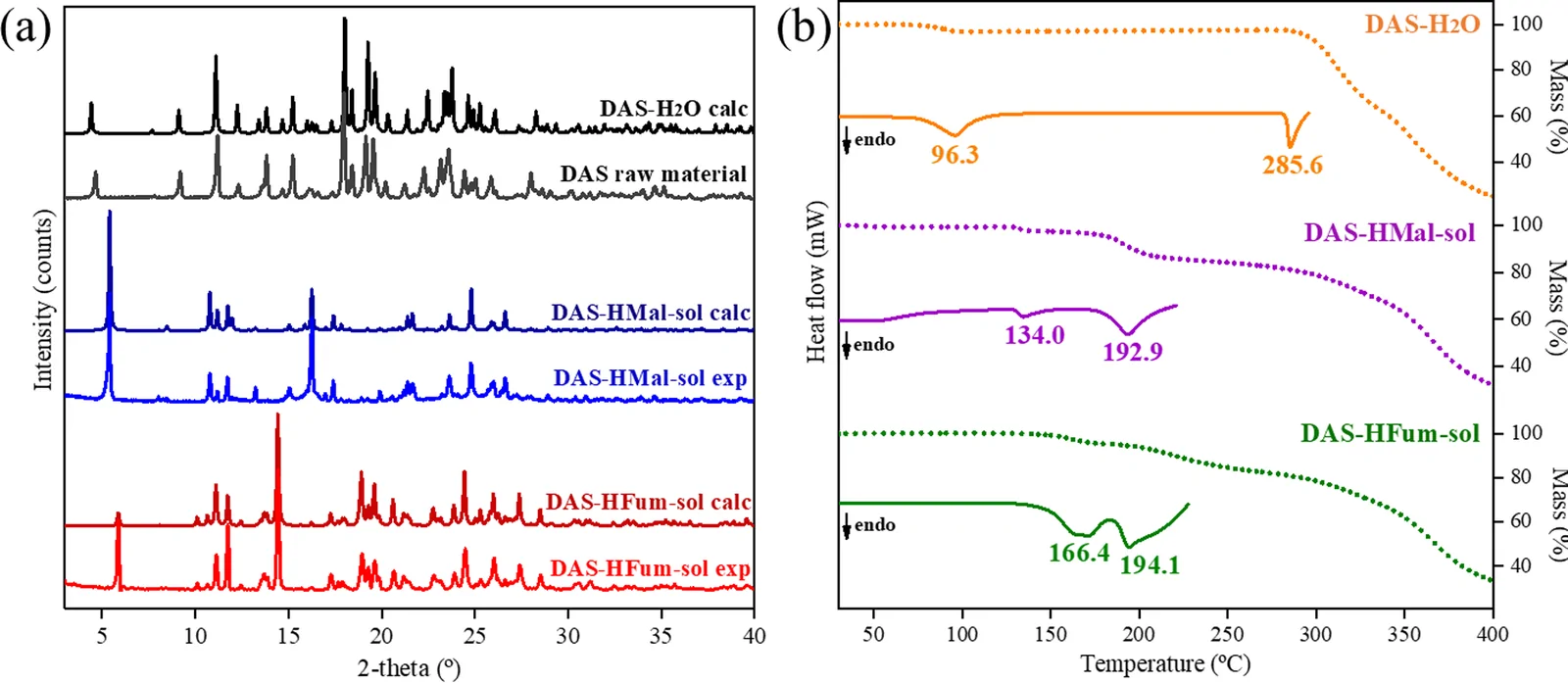
(a) Experimental (exp) and calculated (calc) diffractograms and (b) DSC curves (solid lines) and TG thermograms (dashed lines) for DAS solid forms.

### 3.5 Thermal Characterization

The thermal profile of DAS solid forms was revealed by DSC and TG, as shown in Figure 4b. DSC and TG thermograms of the DAS commercial monohydrate form were included for comparison. The DSC curve of parent DAS exhibits two well-defined endothermic peaks at 96.3 °C (*T*_onset_ = 73.01 °C) and 285.6 °C (*T*_onset_ = 281.50 °C) assigned to the dehydration and melting/degradation processes of the API, respectively. The early peak has been accompanied by an initial mass loss of approximately 3.1% at around 100 °C on the TG curve, consistent with the release of one water molecule from the crystal structure. The second melting/degradation peak correlated with a gradual mass loss in the TG curve after 287 °C. After dehydration, DAS remains stable in the anhydrous form.^42^ The new solvated salts DAS-HMal-sol and DAS-HFum-sol exhibited distinct thermal behavior compared to pure DAS, reflecting their more diverse and less cohesive salt crystal packing.

The thermal profiles of DAS-HMal-sol and DAS-HFum-sol salt solvates are similar, showing two endothermic peaks in DSC curves and a gradual thermal decomposition in TG thermograms after ∼180 °C. The first DSC peak is assigned to the salt desolvation, while the second one (between ∼192–194 °C) is attributed to the salt melting/degradation process. For DAS-HMal-sol, it is noted that residual solvent molecules, specifically ethanol, remaining in accessible voids of the structure are released close to 134.0 °C, as evidenced by the discrete endothermic event and mass loss at ∼2.5% in the DSC and TG curves, respectively. As previously demonstrated in the crystallography and ^1^H NMR spectroscopy sections, ethanol molecules are not only present in the DAS-HMal-sol crystal structure but also fill, nonhomogeneously, part of the solvent-accessible voids, being consistent with a nonstoichiometric solvated salt (1:1:0.35, drug:coformer:solvent). For DAS-HFum-sol, desolvation occurred only at 166.4 °C since the methanol molecules in the structure are strongly H-bonded to fumarate anions. In this temperature range, a mass loss of ∼4.5% is observed in the corresponding TG curve. This value corresponds to the release of approximately one molecule of methanol from the crystal structure, which aligns with the stoichiometry of 1:1:1 (drug:coformer:solvent), which provides a solvent weight loss theoretical value of about 5.0%. Therefore, based on the results from the DSC and TG curves and degradation onset temperatures, the following order of thermal stability for DAS solid forms has been established: DAS-H_2_O > DAS-HFum-sol ≈ DAS-HMal-sol.

### 3.6 Equilibrium Solubility and Intrinsic Dissolution Profiles

Aqueous solubility is a crucial property of solid drug products, significantly affecting the bioavailability of an API and, consequently, the pharmacological response.^47^ It is well established that the salt formation strategy readily fine-tunes this attribute, especially for ionizable APIs, and is extensively used for this purpose.^1,3^ For DAS, which displays low and pH-dependent solubility, the synthesis of more soluble salt forms is a primordial requirement for enhancing its oral bioavailability. Thus, the equilibrium solubility plot of unmodified commercial DAS-H_2_O and its reported salt solvate forms in media covering physiological conditions is depicted in Figure 5a. Overall, remarkable improvements in drug solubility were achieved in all dissolution media assessed, achieving our central target. In terms of stability, the new DAS-solvated salts did not significantly affect the pH of the dissolution media after the solubility test. PXRD data revealed that the structural identity of the solid residues and the undissolved disks from the solubility and dissolution experiments remained unchanged, excluding the occurrence of phase transitions and proving the stability of the newly formed solids after dissolution/solubilization. The stability data mentioned are not shown here.

**Figure 5:**
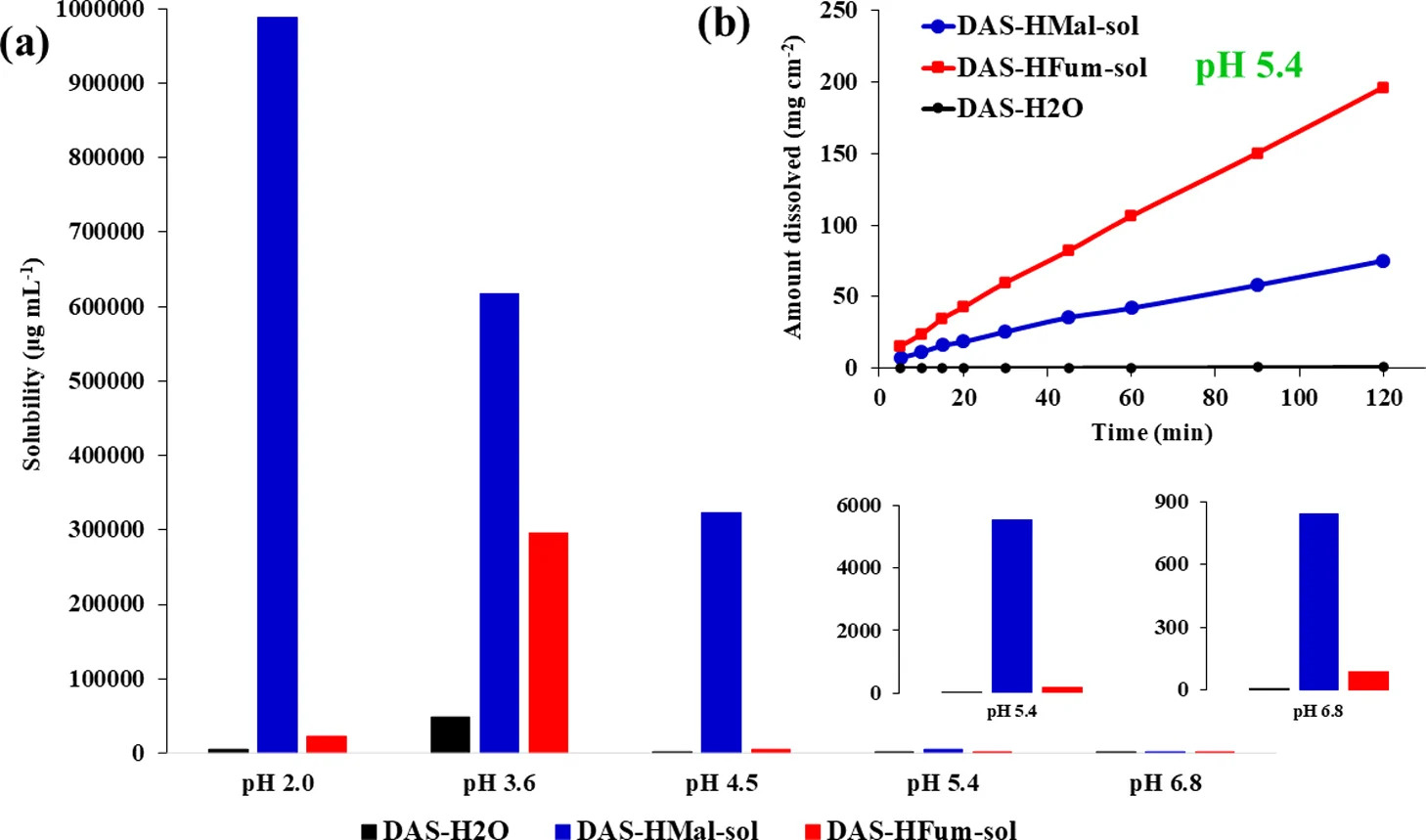
(a) Equilibrium solubility graph of DAS solid forms in different dissolution media. (b) Intrinsic dissolution profile of DAS solid forms in phosphate buffer pH 5.4.

Indeed, DAS solubility is very low and pH-dependent, consistent with data described in the literature^16^ with the highest values in acidic media. The new DAS-HMal-sol and DAS-HFum-sol salt solvates, which followed this pH-dependent solubility trend, achieved solubilities that were remarkably superior across all dissolution media evaluated (Table 2). Corroborating our expectations, DAS-HMal-sol showed a significant API solubility improvement, reaching an impressive 1470-fold increase at pH 4.5. At pH values of 5.4 and 6.8, DAS-HMal-sol was approximately 465 and 838 times more soluble than DAS-H_2_O. Although more discretely, DAS-HFum-sol also provided considerable enhancements in the solubility of DAS in all dissolution media, being around 85 times more soluble compared to the parent drug at pH 6.8. Hence, the following solubility order can be established: DAS-HMal-sol > DAS-HFum-sol > DAS-H_2_O.

**Table 2:** Equilibrium Solubilities and IDRs of DAS Solid Forms^a^

solid form	solubility (μg mL^–1^)					IDR (mg cm^–2^ min ^–1^) pH 5.4
	pH 2.0	pH 3.4	pH 4.5	pH 5.4	pH 6.8	
DAS-H_2_O	5041.5	48402.3	220.0	11.9	1.0	0.005
DAS-HMal-sol	987753.5	617884.8	323315.4	5543.3	838.5	0.572
DAS-HFum-sol	22478.5	295493.2	4616.9	165.1	85.4	1.558

a IDR: intrinsic dissolution rate.

In addition to conducting solubility studies, we also determined the intrinsic dissolution rates (IDRs) of the DAS solid forms to establish the effect of the solid-state changes on drug dissolution kinetics. The pH 5.4 chosen closely resembles the pH of the intestinal portion, where DAS is most absorbed *in vivo*.^48^ The new salt solvate forms of maleate and fumarate presented more favorable IDRs than hydrated DAS (Table 2). The DAS-HMal-sol (0.572 mg cm^–2^ min^–1^) and DAS-HFum-sol (1.558 mg cm^–2^ min^–1^) exhibited a 114- and 312-fold faster dissolution rate, respectively, compared to DAS-H_2_O (0.005 mg cm^–2^ min^–1^), as illustrated in Figure 5b. Surprisingly, the DAS-HFum-sol salt solvate possibly exhibited a further “spring-parachute” effect^49^ in the initial hours of intrinsic dissolution experiments, and this eventual metastability of the dissolved species can justify the very high IDR found for this salt solvate. Accordingly, the following IDR order can be established: DAS-HFum-sol > DAS-HMal-sol > DAS-H_2_O.

Both solubility and intrinsic dissolution tests revealed a remarkable increase in drug solubility upon salt formation, and this can be correlated with some crystallographic features of these solvated salts. The solid-state arrangement of DAS with maleate and fumarate counterions derived from the coformers, rich in atoms O and highly avid for hydrogen bonds, enhances the occurrence of polar supramolecular synthons and contributes to the emergence of hydrophilic regions in the crystal structures. From an energetic framework point of view, the insertion of coformers into the crystal lattice generates less efficient 3D arrangements and, consequently, less energetically stable ones (see next Section 3.8). These combined solid-state attributes justify, in part, the higher IDRs and water solubility of these new DAS salt solvates compared with the DAS neutral form. This result confirms that the salt formation of DAS has a noteworthy influence on the solubility and IDR of the API. Further, it indicates that both solvated salts tend to readily solubilize in the medium mimicking the physiological one, which may favor drug absorption.

### 3.7 Stability Outcomes

In addition to improved solubility and dissolution, the DAS crystal forms also demonstrated high solid-state stability. Stability data for newly synthesized and unstressed DAS solid forms compared to samples exposed to accelerated degradation conditions are summarized in Table 3. All initial samples have a high purity of almost 100% of DAS. The DAS solvated salt forms retained almost entirely their original form after 5 days of exposure to UV radiation, proving that solvated salts DAS-HMal-sol and DAS-HFum-sol are also highly photostable. Resemble stability results have been found after DAS solid samples are maintained for 180 days at 40 °C and 75% RH, indicating that high temperature and relative humidity have low degradative potential for the samples.

**Table 3:** DAS Levels Found in Freshly Prepared and Accelerated-Degradation DAS Solid Samples

solid form	freshly prepared (initial)	after 5 days under light stress	after 180 days at 40 °C and 75% RH
	% DAS	% DAS	% DAS
DAS-H_2_O	98.3 ± 0.3	97.9 ± 0.3	97.3 ± 0.2
DAS-HMal-sol	98.4 ± 0.2	96.3 ± 0.2	97.1 ± 0.2
DAS-HFum-sol	99.0 ± 0.2	96.5 ± 0.2	97.4 ± 0.3

### 3.8 Energy Framework Analysis

To understand the energetic contributions of supramolecular motifs in stabilizing interaction forces in DAS MCCs, energy framework studies have been utilized. Figures S6–S8 depict the relative magnitude, and Figure 6 illustrates the topology of the interaction energies for DAS solid forms. The DAS^+^ cation, the counterparts maleate/fumarate anions, and solvent/water molecules are stabilized by both Coulombian (red) and dispersive (green) energies. Solvent molecules (water, methanol, and ethanol) have been considered in the calculations to evaluate their contribution to crystal lattice stabilization. For DAS-HMal, EF calculations conducted both with and without the solvent—specifically, disordered ethanol in the solvent-accessible structural channels—produced very similar results. This suggests that, for this particular salt structure, the solvent has a minimal effect on the crystal lattice stabilization. Due to the difference in supramolecular architectures, each DAS MCC exhibits a unique and irregular energy topology in the solid state. Nevertheless, a thorough examination of the stabilizing interaction magnitude for each MCC may clarify the reason for the high aqueous solubility and lower thermal stability of DAS after its salt formation. It is well established that crystal structures efficiently packed are more thermally stable. On the other hand, the aqueous solubility of the API is generally lower.^50^ The DAS commercial form corroborates this statement; we only need to observe its lower energy framework and high thermal stability (Table 4), besides its low aqueous solubility (Table 2). Following this premise, both DAS-HMal-sol and DAS-HFum-sol forms are significantly less stable in terms of energy framework, and this possibly directly reflects their high aqueous solubilities, rapid IDRs, and low thermal stabilities. Despite the nontriviality between the interaction energy framework and the drug’s physicochemical properties, our results reinforce that aqueous solubility and thermal stability are properties that are prone to being inversely proportional to lattice energy in crystals.

**Figure 6:**
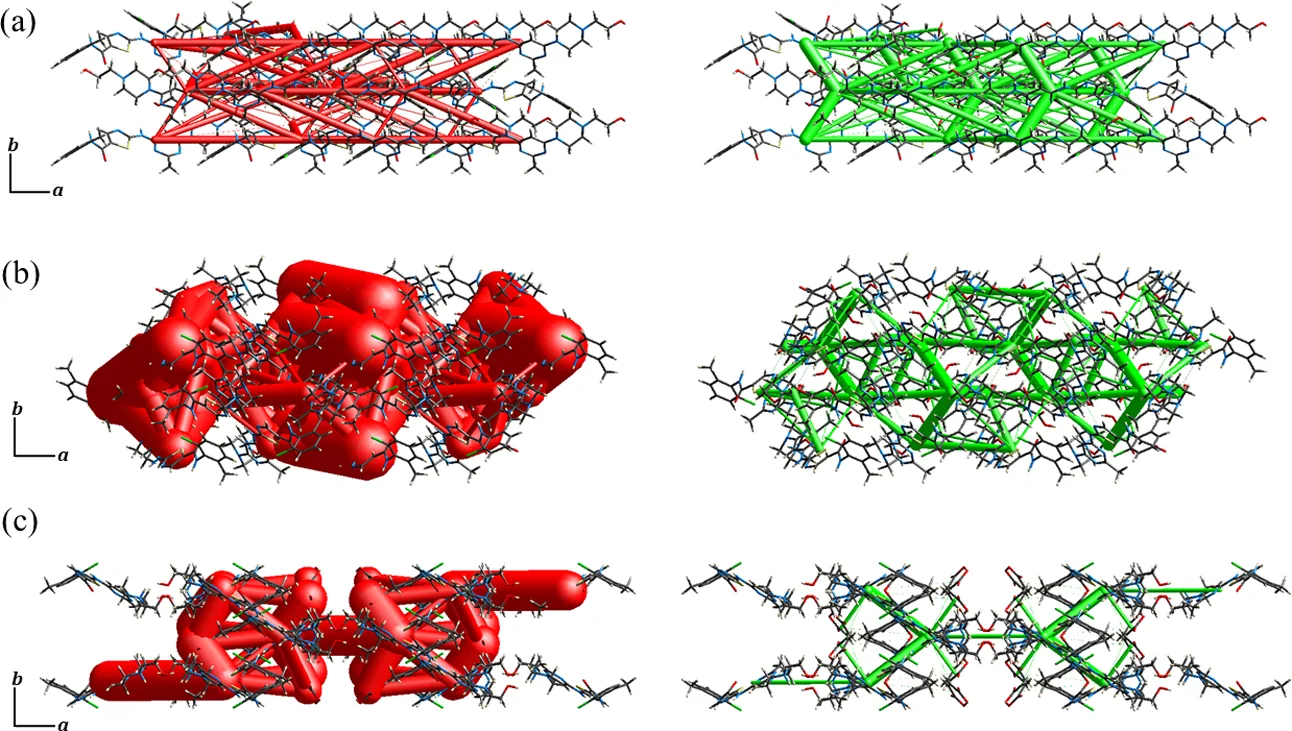
Energy frameworks partitioned into electrostatic (red) and dispersion (green) energetic components for the solids: (a) DAS-H_2_O, (b) DAS-HFum-sol, and (c) DAS-HMal-sol. The values of energy scale factors (tube size) are set to 100.

**Table 4:** Main Thermal and Energetic Data of DAS Solid Samples

solid form	melting/decomposition temp. (degC)	sum of interaction energies (kJ mol^–1^)
DAS-H_2_O	285.6	–23.85
DAS-HMal-sol	192.9	99.79
DAS-HFum-sol	194.1	92.01

### 3.9 Leukemia Cell Line Viability

The cytotoxicity evaluation of the DAS solid forms in the fibroblast L929 culture demonstrated that none of the MCCs showed toxicity to this cell line. The percentages of cell viability found for the DAS MCCs in all concentrations tested (20–200 μg/mL) and after the three exposure periods (24, 48, and 72 h) were superior to 80%, indicating high cytocompatibility (grade 0, noncytotoxic) according to the International Organization for Standardization (ISO) 10993–5.^51^ In contrast, when human THP-1 leukemia cells were exposed to DAS MCCs, under the same conditions as L929 fibroblasts, we observed significant reductions in cell viability, reaching up to 56% of inhibition at the highest concentrations and over prolonged periods tested. DAS in its native form (DAS-H_2_O) was considerably less effective at inhibiting THP-1 cell viability than its solvated salt forms. Overall, as presented in Figure 7, the new DAS solvated salts are predominantly classified as grade 1 (slightly cytotoxic), with some points reaching grade 2 (moderately cytotoxic) according to ISO 10993–5. This suggests that there are subcytotoxic effects that do not cause appreciable cell death. In summary, the cytotoxicity analysis undertaken indicates a partial selectivity toward tumor cells, suggesting metabolic or cellular stress effects without causing significant apoptosis or necrosis.

**Figure 7:**
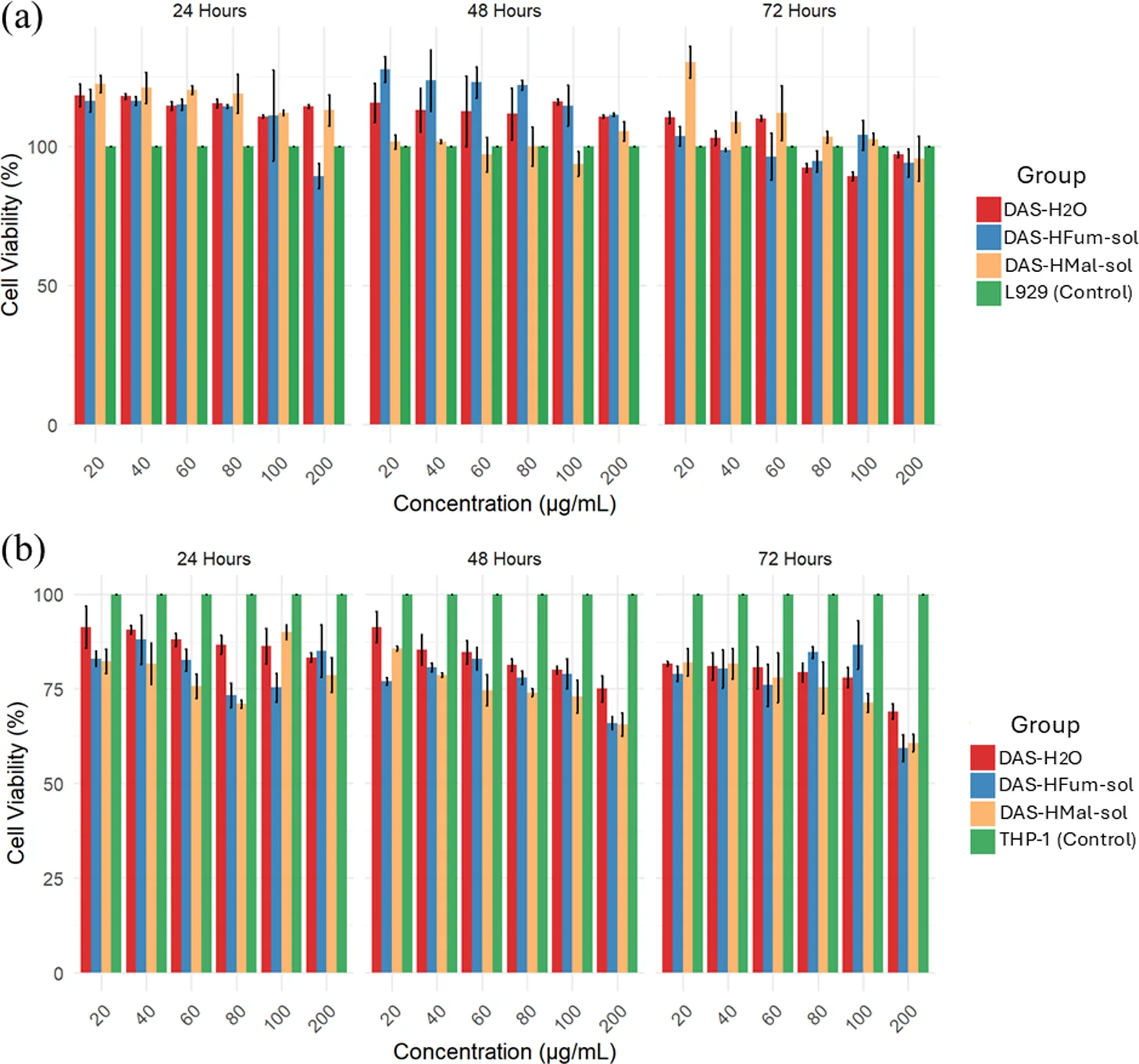
Cell viability percentage of (a) L929 fibroblasts and (b) human THP-1 leukemia cells treated with DAS solid forms.

## 4 Conclusions

A crystalline salt engineering strategy has yielded two MCCs of DAS with the pharmaceutically acceptable maleic and fumaric carboxylic acids. Both DAS-HMal-sol and DAS-HFum-sol forms substantially increased DAS solubility and dissolution rate, making these solvated salts useful and applicable for enhancing poor oral bioavailability. Structural elucidation by SCXRD revealed 3D supramolecular arrangements enriched with polar domains and stabilized mainly by H-bonds. Spectroscopic (FT-IR and ^1^H NMR), PXRD, and thermal (DSC and TG) analyses have indicated the formation of unique and highly pure salt solvates. Moreover, in the solubility and intrinsic dissolution tests, a remarkable improvement in these essential attributes has been achieved by new DAS solid forms. DAS-HMal-sol, for instance, displayed a solubility approximately 1470 times higher at pH 4.5 compared with parent DAS. In terms of cytotoxicity, the DAS MCCs maintained/enhanced the viability of L929 fibroblasts while slightly reducing the cell viability of the human leukemia cell line THP-1, suggesting an interesting selectivity toward tumor cells. Therefore, our investigation demonstrates that the salt formation of DAS with maleic and fumaric acids provides the central requirement for enhancing the oral bioavailability of DAS, i.e., high and rapid solubilization of the API in physiologically like media.

## Supplementary Material



## Notes

ORTEP diagrams, Fourier difference maps, overlay of the DAS molecules, ^1^H NMR spectrum, overview of energy framework calculation, validation results of the chromatographic method, analysis of p*K*_a_ difference, geometric parameters of the principal H-bonds, and FT-IR band assignments (PDF)

## References

[1] Serajuddin A. T. M. (2007). Salt
Formation to Improve Drug Solubility. Adv. Drug
Delivery Rev..

[2] Berry D. J., Steed J. W. (2017). Pharmaceutical Cocrystals,
Salts and Multicomponent
Systems; Intermolecular Interactions and Property Based Design. Adv. Drug Delivery Rev..

[3] Blagden N., de Matas M., Gavan P. T., York P. (2007). Crystal Engineering
of Active Pharmaceutical Ingredients to Improve Solubility and Dissolution
Rates. Adv. Drug Delivery Rev..

[4] Braga D. (2023). Crystal Engineering:
From Promise to Delivery. Chem. Commun..

[5] Bolla G., Sarma B., Nangia A. K. (2022). Crystal
Engineering of Pharmaceutical
Cocrystals in the Discovery and Development of Improved Drugs. Chem. Rev..

[6] Kumar A., Kumar S., Nanda A. (2018). A Review about Regulatory
Status
and Recent Patents of Pharmaceutical Co-Crystals. Adv. Pharm. Bull..

[7] Gadade D. D., Pekamwar S. S. (2016). Pharmaceutical Cocrystals: Regulatory and Strategic
Aspects, Design and Development. Adv. Pharm.
Bull..

[8] Chettri A., Subba A., Singh G. P., Bag P. P. (2024). Pharmaceutical Co-Crystals:
A Green Way to Enhance Drug Stability and Solubility for Improved
Therapeutic Efficacy. J. Pharm. Pharmacol..

[9] Dai X. L., Chen J. M., Lu T. B. (2018). Pharmaceutical
Cocrystallization:
An Effective Approach to Modulate the Physicochemical Properties of
Solid-State Drugs. CrystEngComm.

[10] Elder D. P., Holm R., Diego H. L. d. (2013). Use
of Pharmaceutical Salts and Cocrystals
to Address the Issue of Poor Solubility. Int.
J. Pharm..

[11] Duggirala N. K., Perry M. L., Almarsson O., Zaworotko M. J. (2016). Pharmaceutical
Cocrystals: Along the Path to Improved Medicines. Chem. Commun..

[12] Song W., Wang N., Li A., Ji X., Huang X., Wang T., Hao H. (2024). Multi-Component Crystal Strategy
for Improving Water Solubility and Antifungal Activity of Climbazole. Pharm. Res..

[13] Shimada N., Uchida H., Palanisamy V., Chernyshev V., Fukami T. (2025). Multicomponent Solid Forms of Pioglitazone
and Their
Influence on Drug Dissolution. Cryst. Growth
Des..

[14] Wathoni N., Sari W. A., Elamin K. M., Mohammed A. F. A., Suharyani I. (2022). A Review of
Coformer Utilization in Multicomponent Crystal Formation. Molecules.

[15] Tokarski J. S., Newitt J. A., Chang C. Y. J., Cheng J. D., Wittekind M., Kiefer S. E., Kish K., Lee F. Y. F., Borzillerri R., Lombardo L. J., Xie D., Zhang Y., Klei H. E. (2006). The Structure
of Dasatinib (BMS-354825) Bound to Activated ABL Kinase Domain Elucidates
Its Inhibitory Activity against Imatinib-Resistant ABL Mutants. Cancer Res..

[16] Eley T., Luo F. R., Agrawal S., Sanil A., Manning J., Li T., Blackwood-Chirchir A., Bertz R. (2009). Phase I Study of the
Effect of Gastric Acid PH Modulators on the Bioavailability of Oral
Dasatinib in Healthy Subjects. J. Clin. Pharmacol..

[17] Keerikkadu M., Bangera P. D., Tippavajhala V. K., Rathnanand M. (2025). Dasatinib
Pharmacokinetics and Advanced Nanocarrier Strategies: From Systemic
Limitations to Targeted Success. AAPS PharmSciTech.

[18] Levêque D., Becker G., Bilger K., Natarajan-Amé S. (2020). Clinical Pharmacokinetics
and Pharmacodynamics of Dasatinib. Clin. Pharmacokinet..

[19] Abdul
Azeeze M. S. T., Kandasamy S., Palanisamy P., Palanichamy P., Subramania Nainar M. (2023). Cocrystallization of Dasatinib with
Different Acids Improves the Solubility and Physicochemical Properties:
A Combined Experimental and Theoretical Studies. Chemical Physics Impact.

[20] Mondal P. K., Mirmehrabi M., Rohani S. (2021). Insight into the Formation of Heteromolecular
Hydrogen Bonds between Dasatinib and GRAS Molecules. Org. Process Res. Dev..

[21] Cutrignelli A., Sanarica F., Lopalco A., Lopedota A., Laquintana V., Franco M., Boccanegra B., Mantuano P., De Luca A., Denora N. (2019). Dasatinib/HP-β-CD
Inclusion Complex Based Aqueous
Formulation as a Promising Tool for the Treatment of Paediatric Neuromuscular
Disorders. Int. J. Mol. Sci..

[22] Wang C., Wang M., Chen P., Wang J., Le Y. (2022). Dasatinib
Nanoemulsion and Nanocrystal for Enhanced Oral Drug Delivery. Pharmaceutics.

[23] Dharani S., Mohamed E. M., Khuroo T., Rahman Z., Khan M. A. (2022). Formulation
Characterization and Pharmacokinetic Evaluation of Amorphous Solid
Dispersions of Dasatinib. Pharmaceutics.

[24] Mageshvaran D., Yadav S., Yadav V., Kuche K., Katari O., Jain S. (2025). Enhancing Oral Bioavailability
of Dasatinib via Supersaturable SNEDDS:
Investigation of Precipitation Inhibition and IVIVC through in-Vitro
Lipolysis-Permeation Model. Int. J. Pharm..

[25] Shaikh R., Bhattacharya S., Saoji S. D. (2024). Development, Optimization,
and Characterization
of Polymeric Micelles to Improve Dasatinib Oral Bioavailability: Hep
G2 Cell Cytotoxicity and in Vivo Pharmacokinetics for Targeted Liver
Cancer Therapy. Heliyon.

[26] Zhou X., Zhuang Y., Liu X., Gu Y., Wang J., Shi Y., Zhang L., Li R., Zhao Y., Chen H., Li J., Yao H., Li L. (2023). Study on Tumour Cell-Derived Hybrid
Exosomes as Dasatinib Nanocarriers for Pancreatic Cancer Therapy. Artif. Cells Nanomed. Biotechnol..

[27] Sarceviča I., Grante I., Belyakov S., Rekis T., Běrziņš K., Actiņš A., Orola L. (2016). Solvates of Dasatinib: Diversity
and Isostructurality. J. Pharm. Sci..

[28] Trimdale-Deksne A., Grante I., Mishnev A., Orola L., Be̅rziṇš A. (2024). Similarity
and Differences of Dasatinib Solvates: A Crystallographic Perspective. Cryst. Growth Des..

[29] Saladi V. N., Kammari B. R., Maruthapillai A., Mahapatra S., Chennuru R., Sajja E., Rajan S. T., Mathad V. T. (2022). Stable
Fatty Acid Solvates of Dasatinib, a Tyrosine Kinase Inhibitor: Prediction,
Process, and Physicochemical Properties. ACS
Omega.

[30] Dolomanov O. V., Bourhis L. J., Gildea R. J., Howard J. A. K., Puschmann H. (2009). OLEX2: A Complete
Structure Solution, Refinement and Analysis Program. J. Appl. Crystallogr..

[31] Sheldrick G. M. (2015). SHELXT
- Integrated Space-Group and Crystal-Structure Determination. Acta Crystallogr. A.

[32] Sheldrick G. M. (2015). Crystal
Structure Refinement with SHELXL. Acta Crystallogr.
C Struct. Chem..

[33] Spek A. L. (2015). PLATON
SQUEEZE: A Tool for the Calculation of the Disordered Solvent Contribution
to the Calculated Structure Factors. Acta Crystallogr.
C Struct. Chem..

[34] Macrae C. F., Sovago I., Cottrell S. J., Galek P. T. A., McCabe P., Pidcock E., Platings M., Shields G. P., Stevens J. S., Towler M., Wood P. A. (2020). Mercury
4.0: From Visualization to
Analysis, Design and Prediction. J. Appl. Crystallogr..

[35] Spackman P. R., Turner M. J., McKinnon J. J., Wolff S. K., Grimwood D. J., Jayatilaka D., Spackman M. A. (2021). CrystalExplorer: A Program for Hirshfeld
Surface Analysis, Visualization and Quantitative Analysis of Molecular
Crystals. J. Appl. Crystallogr..

[36] Groom C. R., Bruno I. J., Lightfoot M. P., Ward S. C. (2016). The Cambridge Structural
Database. Acta Crystallogr. B Struct. Sci. Cryst.
Eng. Mater..

[37] ICH ICH Topic Q2 (R1) Validation of Analytical Procedures; Text and Methodology: Geneva, 2005.

[38] Glomme A., März J., Dressman J. B. (2005). Comparison of a
Miniaturized Shake-Flask
Solubility Method with Automated Potentiometric Acid/Base Titrations
and Calculated Solubilities. J. Pharm. Sci..

[39] ICH ICH Topic Q1B Photostability Testing of New Active Substances and Medicinal Products Step 5 NOTE FOR GUIDANCE ON THE PHOTOSTABILITY TESTING OF NEW ACTIVE SUBSTANCES AND MEDICINAL PRODUCTS, 1998.

[40] Cruz-Cabeza A. J. (2012). Acid–Base
Crystalline Complexes and the PKa Rule. CrystEngComm.

[41] Cruz-Cabeza A. J., Lusi M., Wheatcroft H. P., Bond A. D. (2022). The Role of Solvation
in Proton Transfer Reactions: Implications for Predicting Salt/Co-Crystal
Formation Using the ΔpKa Rule. Faraday
Discuss..

[42] Roy S., Quiñones R., Matzger A. J. (2012). Structural and Physicochemical Aspects
of Dasatinib Hydrate and Anhydrate Phases. Cryst.
Growth Des..

[43] Heinz A., Strachan C. J., Gordon K. C., Rades T. (2009). Analysis of Solid-State
Transformations of Pharmaceutical Compounds Using Vibrational Spectroscopy. J. Pharm. Pharmacol..

[44] Qin Q. P., Meng T., Tan M. X., Liu Y. C., Luo X. J., Zou B. Q., Liang H. (2018). Synthesis Crystal Structure and Biological
Evaluation of a New Dasatinib Copper­(II) Complex as Telomerase Inhibitor. Eur. J. Med. Chem..

[45] Kim Y. R., Kim Y. H., Kim S. W., Lee Y. J., Chae D. E., Kim K. A., Lee Z. W., Kim N. D., Choi J. S., Choi I. S., Lee K. B. (2016). A Bioorthogonal Approach for Imaging
the Binding between Dasatinib and Its Target Proteins inside Living
Cells. Chem. Commun..

[46] Thakral N. K., Zanon R. L., Kelly R. C., Thakral S. (2018). Applications of Powder
X-Ray Diffraction in Small Molecule Pharmaceuticals: Achievements
and Aspirations. J. Pharm. Sci..

[47] Williams H. D., Trevaskis N. L., Charman S. A., Shanker R. M., Charman W. N., Pouton C. W., Porter C. J. (2013). Strategies to Address Low Drug Solubility
in Discovery and Development. Pharmacol. Rev..

[48] Furmanski B. D., Hu S., Fujita K. I., Li L., Gibson A. A., Janke L. J., Williams R. T., Schuetz J. D., Sparreboom A., Baker S. D. (2013). Contribution of ABCC4-Mediated Gastric
Transport to
the Absorption and Efficacy of Dasatinib. Clin.
Cancer Res..

[49] Bavishi D. D., Borkhataria C. H. (2016). Spring and Parachute: How Cocrystals Enhance Solubility. Prog. Cryst. Growth Charact. Mater..

[50] Freitas J. T. J., Diniz L. F., Gomes D. S., de Paula P. M. A. F., de Castro S. H. A., Martins L. S., Silva D. F., Horta A. L. M., Guimarães F. A.
S., Calisto V. F. M., Diniz R. (2022). Energy Framework
and Solubility: A New Predictive Model in the Evaluation of the Structure-Property
Relationship of Pharmaceutical Solid Forms. CrystEngComm.

[51] ISO 10993–5 ISO International Organization for Standardization Biological of Medical Devices - Part 5; Tests for in Vitro Cytotoxicity: Geneva, Switzerland, 2009.

