# Supplementary material for: Fast-Dissolving and Highly Soluble Dasatinib Multicomponent Crystals Efficiently Inhibit Leukemia Cell Viability
Source: ACS Omega. 2026 Sep 19;11(39):58686–97. doi: 10.1021/acsomega.6c01923 (PMC13647392; doi:10.1021/acsomega.6c01923)
Supplement: Supplementary file 1 [file ao6c01923_si_001.pdf]

ELECTRONIC SUPPLEMENTARY INFORMATION

Fast-dissolving and highly soluble dasatinib multicomponent crystals efficiently inhibit leukemia cell viability

Luan F. Diniz[a], Luiz. C. G. Diógenes[a], Paulo S. Carvalho Jr[b], Ezequiel A. D. Junior[a], Alexia D. Nascimento[c], Maria E. Cortés[c], Rubén Dario Sinisterra[d], Renata Diniz[d], Christian Fernandes[a*]*

[a]Departamento de Produtos Farmacêuticos, Faculdade de Farmácia, Universidade Federal de Minas Gerais, 31270-901, Belo Horizonte, MG, Brazil.

[b]Instituto de Física, Universidade Federal do Mato Grosso do Sul, 79074-460, Campo Grande, MS, Brazil.

[c]Departamento de Odontologia Restauradora, Faculdade de Odontologia, Universidade Federal de Minas Gerais, 31270-901, Belo Horizonte, MG, Brazil.

[d]Departamento de Química, Instituto de Ciências Exatas, Universidade Federal de Minas Gerais, 31270-901, Belo Horizonte, MG, Brazil.

*Corresponding Authors: luandiniz@ufmg.br; cfernandes@farmacia.ufmg.br

COMPLEMENTARY FIGURES AND TABLES

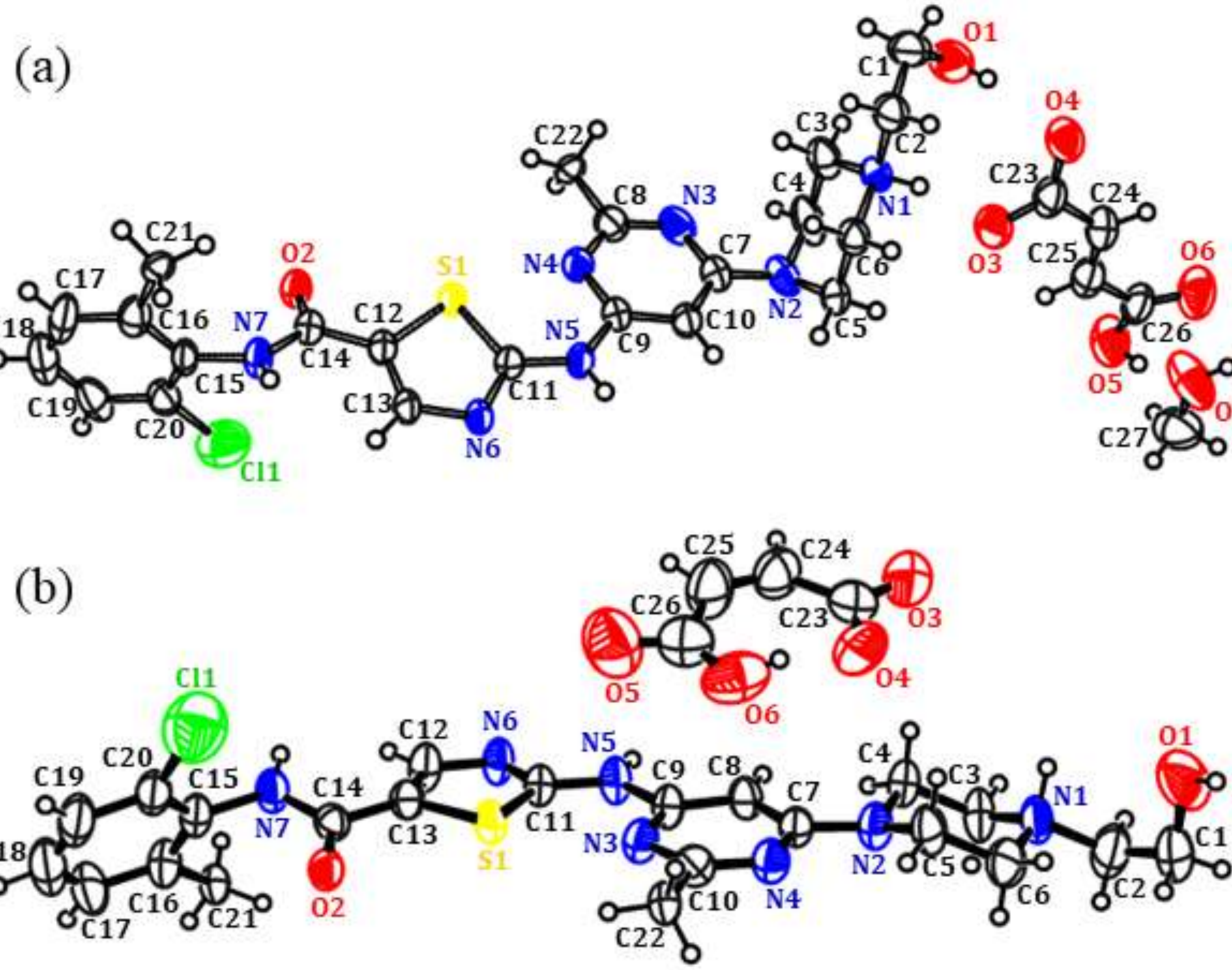

Figure S1. ORTEP diagrams of the DAS salts solvated asymmetric units with 50% probability ellipsoids showing atomic numbering scheme: (a) dasatinib fumarate (DAS-HFum-sol), (b) dasatinib maleate (DAS-HMal-sol) by applying the SQUEZZE procedure. Hydrogen atoms are shown as spheres of arbitrary radii.

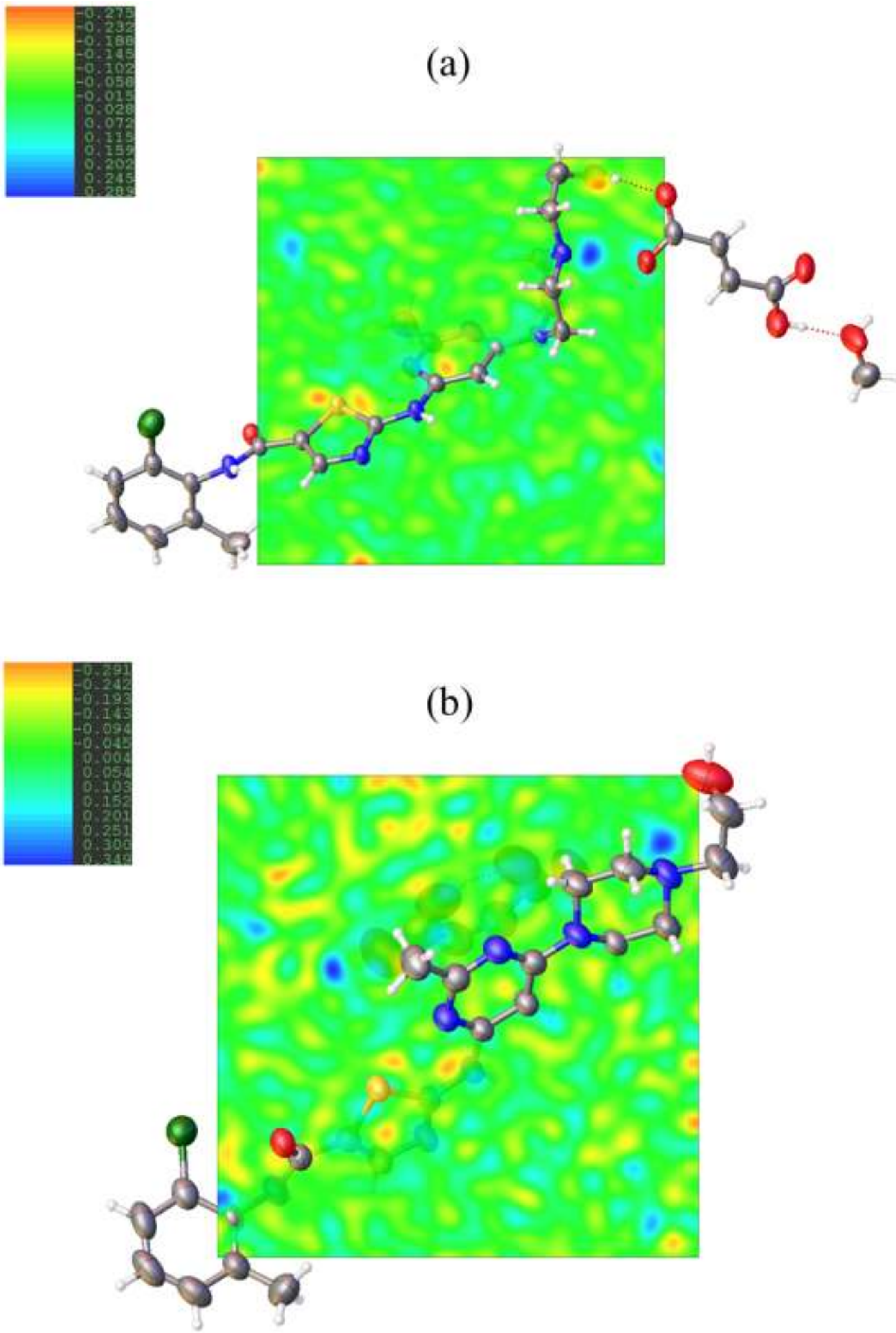

Figure S2. Fourier difference maps of (a) DAS-HFum-sol and (b) DAS-HMal-sol salts solvate, under different perspectives, showing in which N-atoms the H-atoms are attached (blue spots).

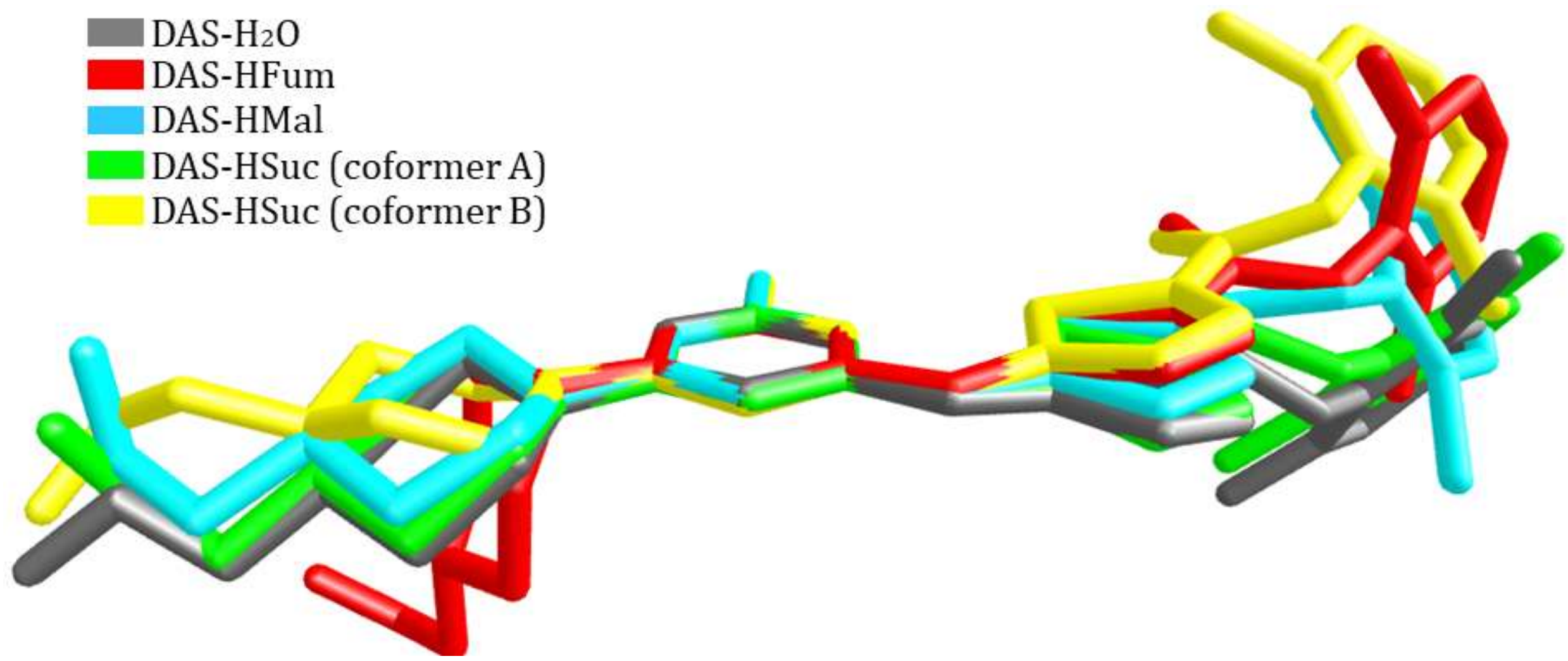

Figure S3. Overlay of the DAS molecules from the commercial monohydrate form (grey), DAS-HFum salt solvate (red), DAS-HMal salt solvate (light blue), DAS-HSuc coformer A (green), and DAS-HSuc coformer B (yellow), using the pyrimidine moieties as the common reference.

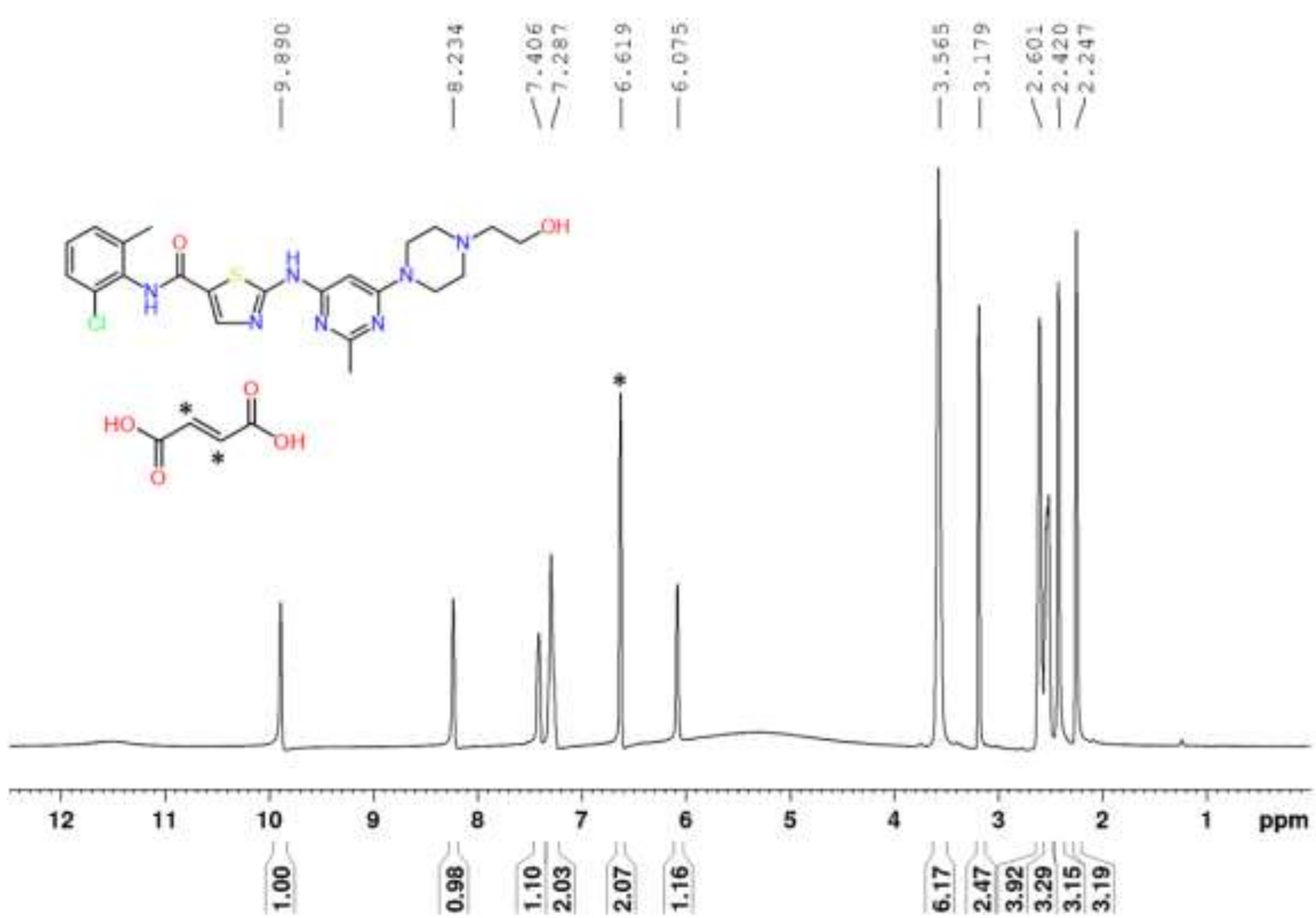

Figure S4. ^{1}H NMR (400 MHz) spectrum of dasatinib fumarate salt solvate (DAS-HFum-sol) in DMSO-d_6.

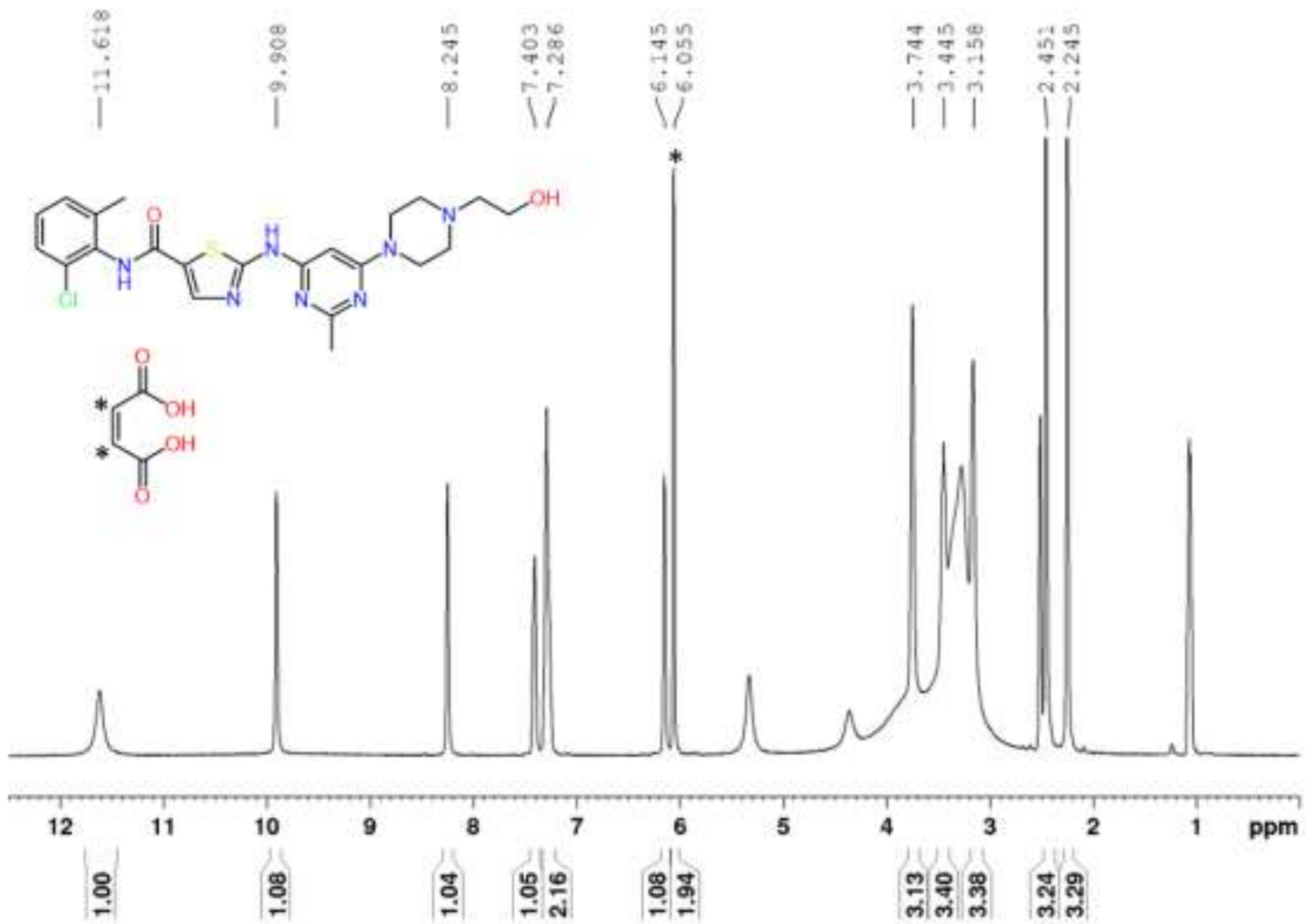

Figure S5. ^{1}H NMR (400 MHz) spectrum of dasatinib maleate salt solvate (DAS-HMal-sol) in DMSO-d_6.

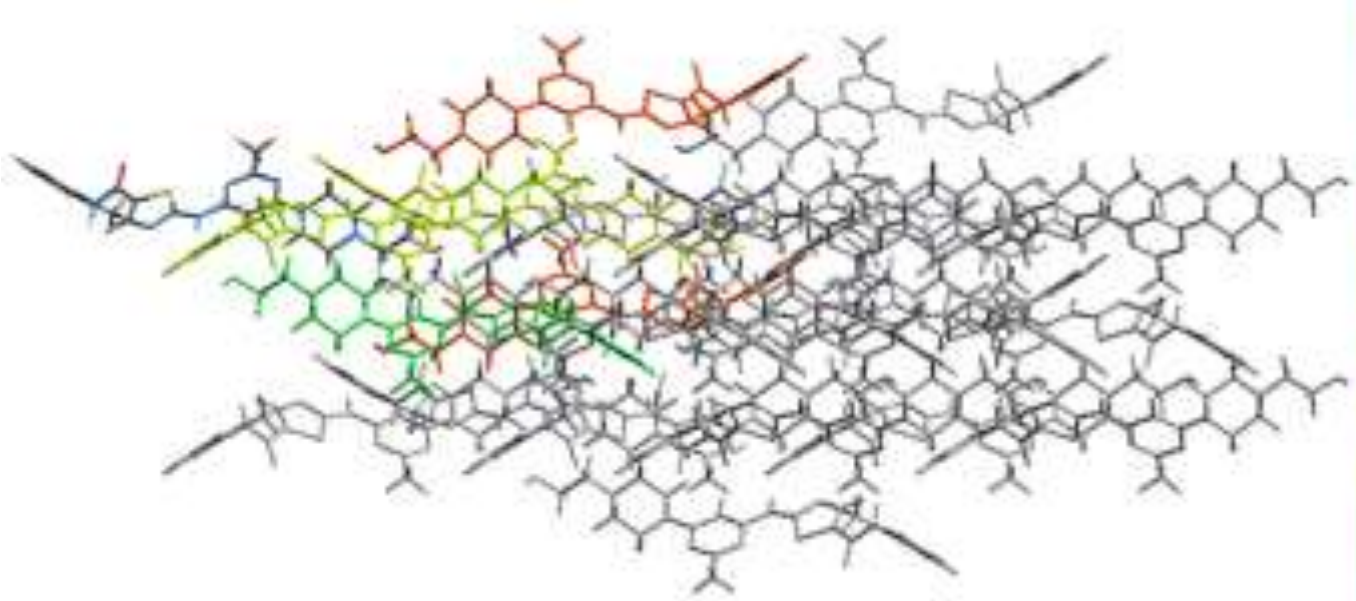

[B3LYP/6-31G(d,p)]

Symop	R	E_ele	E_pol	E_dis	E_rep	E_tot
-	9.06	-38.2	-10.2	-18.2	54.3	-30.3
-	13.83	-31.9	-6.5	-4.5	0.0	-42.4
-	4.79	-34.5	-8.5	-8.5	35.5	-28.2
-	7.38	-1.6	-1.5	-5.7	6.5	-3.7
-	9.24	-0.4	-0.3	-1.7	0.3	-2.0
-x, -y, -z	3.39	-8.8	-0.6	-1.9	1.1	-10.7
-	14.94	-1.3	-0.5	-1.3	0.0	-2.8
-	7.38	-1.6	-1.5	-5.7	6.5	-3.7
-x+1/2, y+1/2, z	19.79	0.0	-1.7	-13.9	0.0	-13.3
-x+1/2, y+1/2, z	7.08	-16.4	-4.3	-74.0	47.4	-55.7
-x, y+1/2, -z+1/2	10.28	0.7	-0.3	-9.3	5.1	-4.4
x, y, z	13.87	-34.6	-13.0	-33.4	0.0	-75.3
x+1/2, -y+1/2, -z	12.16	-4.5	-1.1	-15.8	0.0	-19.4
-	13.83	-29.3	-6.5	-4.5	0.0	-39.6
-	9.06	-38.2	-10.2	-18.2	54.3	-30.3
-x, -y, -z	16.84	-16.6	-2.2	-28.3	0.0	-43.8
-	4.79	-34.5	-8.5	-8.5	35.5	-28.2
-	9.24	-0.4	-0.3	-1.7	0.3	-2.0
x+1/2, y, -z+1/2	11.40	-10.9	-2.4	-28.4	21.4	-24.8
-x+1/2, y+1/2, z	9.65	-35.7	-11.9	-39.2	52.4	-48.4
-x, y+1/2, -z+1/2	18.46	-3.1	-0.6	-11.0	0.0	-13.3
-	14.94	-0.7	-0.5	-1.3	0.0	-2.3

Figure S6. Interaction energies for the DAS hydrated form calculated with the B3LYP model. *R* is the distance (Å) between molecular centroids (mean atomic position) that appears within a radius of 3.8Å from the DAS$^+$ cation (uncolored molecule).

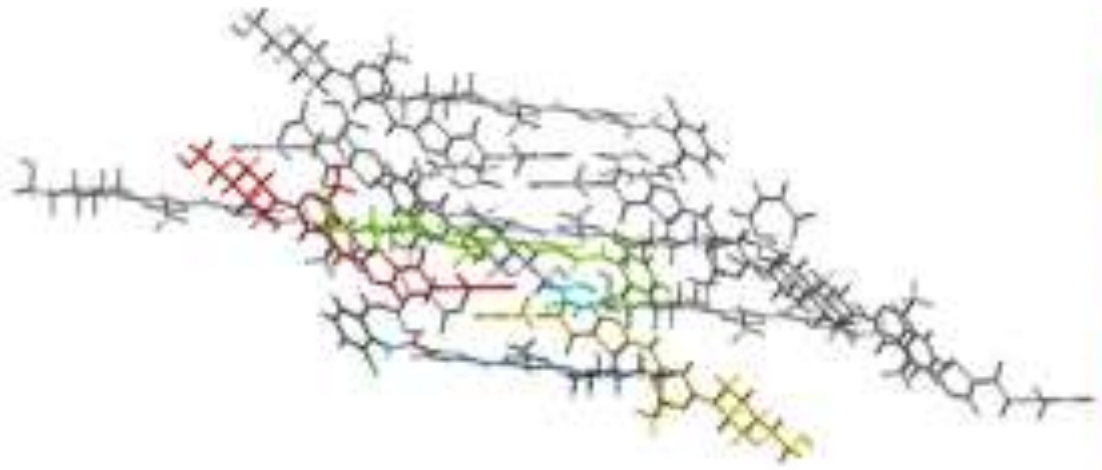

[B3LYP/6-31G(d,p)]

Symop	R	E_ele	E_pol	E_dis	E_rep	E_tot
-x+1/2, y+1/2, -z+1/2	10.43	17.9	-13.7	-64.1	64.8	-7.0
-x+1/2, -y+1/2, -z	6.90	30.5	-49.8	-46.8	114.7	25.5
-x, y, -z+1/2	24.52	185.1	-13.2	-8.0	0.0	178.9
x, -y, z+1/2	11.39	72.4	-32.1	-51.8	32.1	27.5
-x, -y, -z	15.39	185.9	-30.3	-20.1	0.0	156.6
-x+1/2, -y+1/2, -z	7.72	101.0	-21.4	-16.9	5.7	79.9
-	11.83	0.0		0.0	0.0	
-	14.90	0.0	0.0	0.0	0.0	0.0
-x+1/2, -y+1/2, -z	20.43	43.2	-1.2	-15.3	0.0	33.2
-	4.95	185.9	-30.3	-20.1	0.0	156.6
-	7.97	81.6	-2.0	-0.4	0.0	84.4
-	6.99	136.7	-7.7	-1.5	0.0	137.6
-	10.33	66.3	-1.7	-0.3	0.0	68.5
-	13.59	62.2	-1.5	-0.2	0.0	64.4
-	6.99	136.7	-7.7	-1.5	0.0	137.6
-	10.33	66.0	-1.7	-0.3	0.0	68.3
-	13.59	62.3	-1.5	-0.2	0.0	64.5
-	7.97	81.6	-2.0	-0.4	0.0	84.5
-	11.83	0.0		0.0	0.0	
-	4.95	185.9	-30.3	-20.1	0.0	156.6
-	14.90	185.1	-13.2	-8.0	0.0	178.9

Figure S7. Interaction energies for the DAS-HMal-sol salt solvate calculated with the B3LYP model. *R* is the distance (Å) between molecular centroids (mean atomic position) that appears within a radius of 3.8Å from the DAS^+ cation (uncolored molecule).

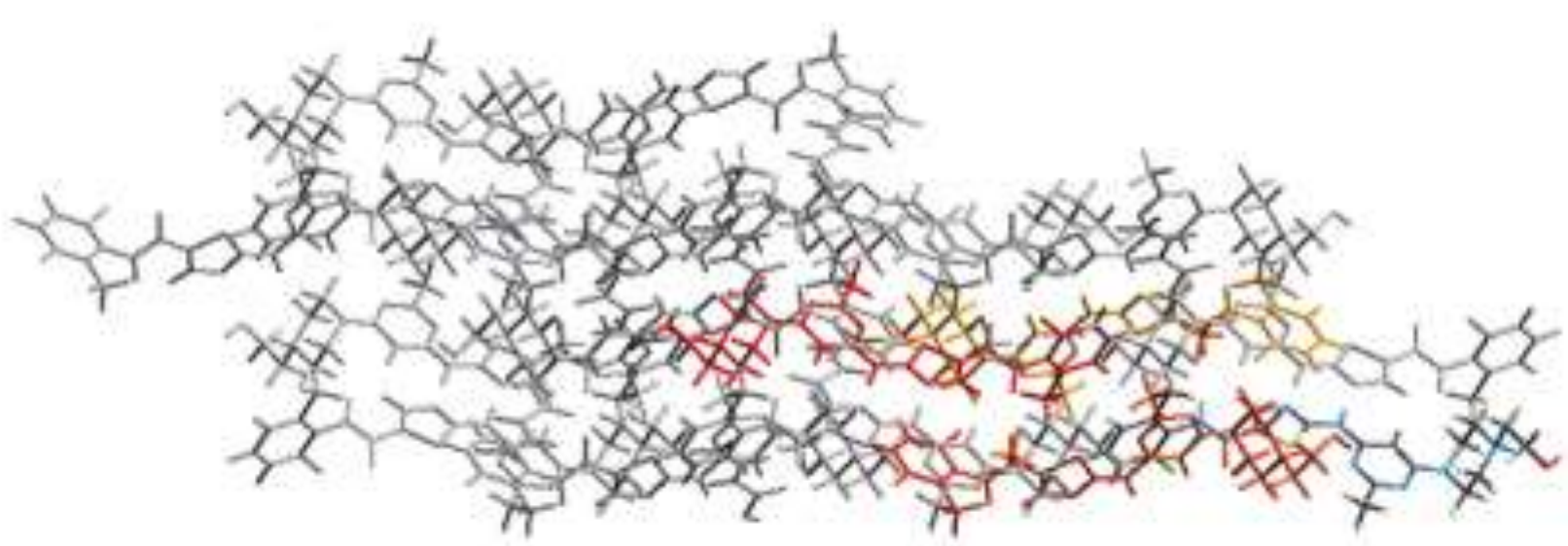

[B3LYP/6-31G(d,p)]

Symop	R	E_ele	E_pol	E_dis	E_rep	E_tot
-x, -y, -z	19.42	46.8	-1.7	-13.0	0.0	36.9
x+1/2, -y+1/2, z+1/2	10.92	55.9	-17.2	-40.8	18.8	64.8
-	8.73	0.0		0.0	0.0	
-	13.10	46.8	-1.7	-13.0	0.0	36.9
-x+1/2, y+1/2, -z+1/2	9.91	24.4	-14.9	-69.3	76.4	3.4
-x, -y, -z	10.63	273.7	-39.0	-13.7	0.0	240.6
-x, -y, -z	7.23	21.4	-59.8	-35.1	89.4	17.9
-	9.12	0.0		0.0	0.0	
-	4.34	0.0	0.0	0.0	0.0	0.0
-	5.99	105.4	-3.8	-1.2	0.0	107.8
-x+1/2, y+1/2, -z+1/2	12.08	135.8	-16.9	-11.5	0.0	121.2
x, y, z	18.81	99.6	-6.9	-9.7	0.0	80.2
-	12.23	63.1	-1.3	-0.2	0.0	65.6
-x, -y, -z	18.74	178.9	-19.6	-13.7	0.0	163.8
-x, -y, -z	7.60	45.3	-9.4	-15.5	1.8	33.4
-	8.21	139.4	-6.8	-2.8	0.0	139.8
-	9.54	82.1	-1.4	-0.1	0.0	85.7
-	6.80	55.9	-17.2	-40.8	18.8	64.8
-	6.38	24.4	-14.9	-69.3	76.4	3.4
-	11.04	273.7	-39.0	-13.7	0.0	240.6
-	10.83	133.5	-16.9	-11.5	0.0	118.7
-	9.12	0.0		0.0	0.0	
-x+1/2, y+1/2, -z+1/2	6.27	133.5	-16.9	-11.5	0.0	118.7
-	4.34	135.8	-16.9	-11.5	0.0	121.2
-	8.21	130.4	-6.8	-2.8	0.0	129.8
-	11.04	273.7	-39.0	-13.7	0.0	240.6
-	12.23	63.1	-1.3	-0.2	0.0	63.6
-	5.70	65.1	-0.7	-0.1	0.0	68.3
-	4.71	105.4	-3.6	-1.2	0.0	107.8
-	9.54	82.3	-1.4	-0.1	0.0	85.8
-	15.10	46.8	-1.7	-13.0	0.0	36.9
-	5.99	106.3	-3.6	-1.2	0.0	108.7
-	6.80	55.9	-17.2	-40.0	18.8	64.5
-	4.71	106.3	-3.6	-1.2	0.0	108.7
-	5.70	65.1	-0.7	-0.1	0.0	68.3
-	10.83	135.8	-16.9	-11.5	0.0	121.2
-	6.38	24.4	-14.9	-69.3	76.4	3.4
-	8.73	0.0		0.0	0.0	

Figure S8. Interaction energies for the DAS-HFum-sol salt solvate calculated with the B3LYP model. *R* is the distance (Å) between molecular centroids (mean atomic position) that appears within a radius of 3.8Å from the DAS^+ cation (uncolored molecule).

Table S1. Results of linearity, accuracy, precision, and limits of quantitation and detection obtained during analytical method validation for DAS.

Parameter	Results
Linearity	
Concentration range (μg mL^{-1})	5.0-25.0
Slope (b)	15563
Intercept (a)	-2725
Coefficient of determination (R^2)	0.9964
Accuracy	
Level 1 (5.0 μg mL^{-1})	Mean recovery: 101.91% (n = 6)
Level 2 (15.0 μg mL^{-1})	Mean recovery: 99.98% (n = 6)
Level 3 (25.0 μg mL^{-1})	Mean recovery: 100.66% (n = 6)
Precision	
Repeatability (RSD%)	1.03-4.72 (n = 3)
Intermediate (RSD%)	0.51-1.23 (n = 6)
Limits	
Quantitation (μg mL^{-1})	0.20
Detection (μg mL^{-1})	0.06

RSD, Relative Standard Deviation.

Table S2. Calculated ΔpKa between DAS and the coformers.

Compound	pKa	ΔpKa = (pKa(base) − pKa(acid))	Stoichiometry (DAS:coformer)
Dasatinib	6.8	-	-
Maleic acid	1.9	4.9	1:1 salt
Fumaric acid	3.0	3.8	1:1 salt

Table S3. Geometric parameters of the principal H-bonds in the DAS MCCs.

Interaction	D···A(Å)	D–H···A(º)	Symmetry Code
		**DAS-HFum-sol**	
O1–H1···O4	2.715(2)	160	*x,y,z*
N1–H1A···O3	2.670(2)	162	*x,y,z*
O5–H5C···O7	2.573(3)	175	*x,y,z*
N5–H5···N6	2.978(2)	169	*-x+1,-y,-z+1*
N7–H7···O2	2.958(2)	172	*-x+1/2,+y-1/2,-z+1/2*
O7–H7A···O4	2.625(3)	165	*-x+1/2+1,+y+1/2,-z+1/2+2*
C13–H13···O2	3.339(2)	130	*-x+1/2,+y-1/2,-z+1/2*
C2–H2B···O6	3.497(3)	148	*x-1/2,-y+1/2,+z-1/2*
C6–H6A···O6	3.445(2)	150	*x-1/2,-y+1/2,+z-1/2*
C4–H4B···O1	3.409(3)	140	*-x+1,-y+1,-z+2*
		**DAS-HMal-sol**	
O1–H1···O6	2.853(8)	137	*-x+1,-y+1,-z*
N5–H5···N6	2.949(8)	177	*-x+1/2+1,-y+1/2,-z+1*
N7–H7···O2	2.818(5)	158	*-x+1/2+1,+y-1/2,-z+1/2+1*
C5–H5B···O4	3.354(8)	136	*x,y,z*
C6–H6A···O4	3.758(7)	167	*-x+1,-y+1,-z*
C12–H12···O12	3.56062)	132	*-x+1/2+1,+y-1/2,-z+1/2+1*
C17–H17···O5	3.353(7)	178	*-x+1/2+1,+y+1/2,-z+1/2+1*
C4–H4B···O2	3.207(8)	128	*x,-y+1,+z-1/2*
C6–H6B···O3	3.420(7)	157	*x,+y+1,+z*

Table S4. Principal FT-IR bands (cm^{-1}) for the novel DAS MCCs.

DAS	DAS-HMal-sol	DAS-HFum-sol	Assignment
3205	3207	3215	ν(N–H)
2955, 2825	2964, 2869	2970, 2872	$\nu(C–H)_{aliphatic}$
-	1695	1707	$\nu(C=O)_{acid}$
1630	1633	1633	$\nu(C=O)_{amide}$
1612	1616	1618	ν(C=C)
-	1573	1574	$\nu_a(COO)_{carboxylate}$
-	1412	1417	$\nu_s(COO)_{carboxylate}$

ν = stretching; *a* = antisymmetric; *s* = symmetric.
